# Tal1, Gata2a, and Gata3 Have Distinct Functions in the Development of V2b and Cerebrospinal Fluid-Contacting KA Spinal Neurons

**DOI:** 10.3389/fnins.2018.00170

**Published:** 2018-03-29

**Authors:** Livia A. Andrzejczuk, Santanu Banerjee, Samantha J. England, Christiane Voufo, Kadiah Kamara, Katharine E. Lewis

**Affiliations:** Department of Biology, Syracuse University, Syracuse, NY, United States

**Keywords:** CSF-cN, *tal2*, *sox1*, GABA, *pkd2l1*, vsx1, V3, V2c

## Abstract

Vertebrate locomotor circuitry contains distinct classes of ventral spinal cord neurons which each have particular functional properties. While we know some of the genes expressed by each of these cell types, we do not yet know how several of these neurons are specified. Here, we investigate the functions of Tal1, Gata2a, and Gata3 transcription factors in the development of two of these populations of neurons with important roles in locomotor circuitry: V2b neurons and cerebrospinal fluid-contacting Kolmer-Agduhr (KA) neurons (also called CSF-cNs). Our data provide the first demonstration, in any vertebrate, that Tal1 and Gata3 are required for correct development of KA and V2b neurons, respectively. We also uncover differences in the genetic regulation of V2b cell development in zebrafish compared to mouse. In addition, we demonstrate that Sox1a and Sox1b are expressed by KA and V2b neurons in zebrafish, which differs from mouse, where Sox1 is expressed by V2c neurons. KA neurons can be divided into ventral KA″ neurons and more dorsal KA′ neurons. Consistent with previous morpholino experiments, our mutant data suggest that Tal1 and Gata3 are required in KA′ but not KA″ cells, whereas Gata2a is required in KA″ but not KA′ cells, even though both of these cell types co-express all three of these transcription factors. In *gata2a* mutants, cells in the KA″ region of the spinal cord lose expression of most KA″ genes and there is an increase in the number of cells expressing V3 genes, suggesting that Gata2a is required to specify KA″ and repress V3 fates in cells that normally develop into KA″ neurons. On the other hand, our data suggest that Gata3 and Tal1 are both required for KA′ neurons to differentiate from progenitor cells. In the KA′ region of these mutants, cells no longer express KA′ markers and there is an increase in the number of mitotically-active cells. Finally, our data demonstrate that all three of these transcription factors are required for later stages of V2b neuron differentiation and that Gata2a and Tal1 have different functions in V2b development in zebrafish than in mouse.

## Introduction

During development, neural circuits need to be precisely assembled for correct behavioral repertoires to be established. In the ventral spinal cord, several distinct classes of neurons, with particular functional properties, must be specified in correct numbers and locations and make appropriate connections with other neurons and muscle cells for locomotor circuitry to properly function. There are still many unanswered questions about how this occurs. Most studies so far, suggest that the development of distinct spinal neurons is regulated by the transcription factors that each cell type express. For example, some transcription factors control when cells differentiate, others determine the overall identity of the cell and some specify particular functional properties such as axon trajectory, neurotransmitter phenotype, and/or expression of particular neuropeptides (e.g., Moran-Rivard et al., 2001; Gross et al., 2002; Muller et al., 2002; Lanuza et al., 2004; Sapir et al., 2004; Cheng et al., 2005; Pillai et al., 2007; Batista and Lewis, 2008; Hilinski et al., 2016; Juárez-Morales et al., 2016). However, for many classes of spinal neurons, including several of those involved in locomotor circuitry, we still don't know which transcription factors regulate these different aspects of specification and differentiation. In this paper we investigate the functions of Tal1, Gata2a, and Gata3 transcription factors in the development of two classes of ventral spinal neurons with crucial roles in locomotor circuitry: cerebrospinal fluid-contacting Kolmer-Agduhr (KA) neurons (also called CSF-cNs), and V2b neurons.

**Figure 1:**
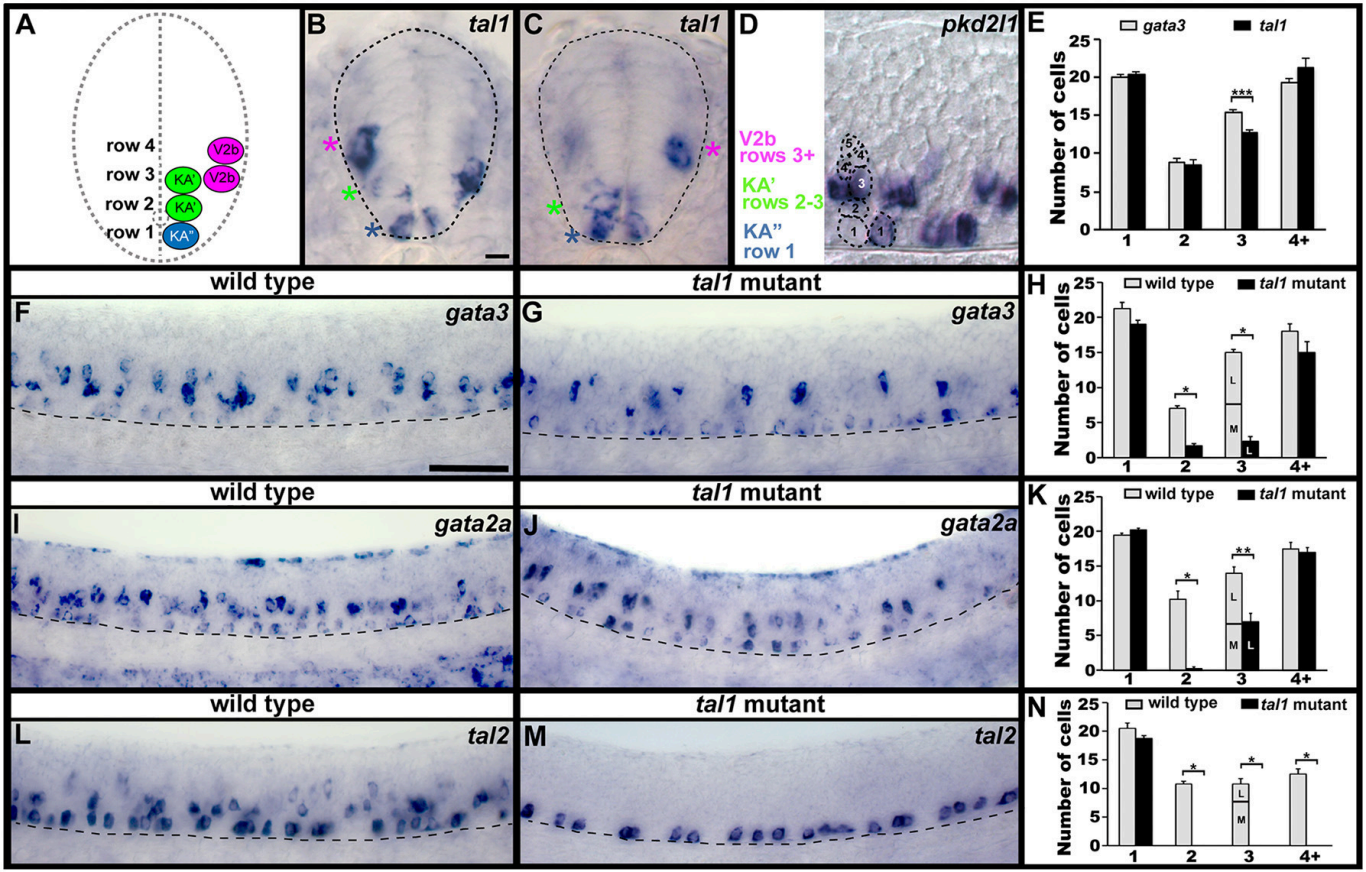
Spinal expression of *tal1* and requirement in KA and V2b neurons. Cross-sectional **(A–C)** and lateral **(D,F,G,I,J,L,M)** views of 24 h zebrafish embryos. Dorsal, top; in lateral views, anterior, left. **(A)** Schematic indicating positions of KA″, KA′, and V2b neurons. **(B,C)** *tal1* expression in KA″ (blue asterisks), KA′ (green asterisks), and V2b (magenta asterisks) cells. **(D)** Example of counting cells in different dorsal/ventral (D/V) “rows” (see section Materials and Methods). Row 3 contains both medial KA′ cells and lateral V2b cells. V2b cells are also located in row 4 and above. **(E,H,K,N)** Mean number of cells expressing specific genes in each D/V row of precisely-defined spinal cord region adjacent to somites 6–10. The approximate proportions of medial and lateral row 3 cells are indicated by horizontal lines separating the number of medially-located cells (bottom and indicated with an “M”) from the number of laterally-located cells (top and indicated with a “L”). All of the remaining *gata3*- and *gata2a*-expressing cells in row 3 of *tal1* mutants were located laterally and were pear shaped, consistent with them being V2b cells, suggesting that no KA′ cells express these genes in *tal1* mutants. *tal1* and *gata3* expression in 24 h WT embryos **(E)**. *gata3* **(F–H)**, *gata2a* **(I–K)**, and *tal2* **(L–N)** expression in WT siblings and *tal1* mutants. Dashed lines indicate spinal cord boundary **(A–C)** or ventral limit of spinal cord **(F,G,I,J,L,M)**. *gata2a* expression ventral to spinal cord and in dorsal trunk is excluded from cell counts **(I)**. Scale bars **(B)** = 10 microns **(B–D)**; **(F)** = 50 microns **(F,G,I,J,L,M)**. All counts were conducted blind to genotype and are an average of at least 4 embryos. Error bars indicate SEM. Statistically significant (*p* < 0.05) comparisons are indicated with brackets and stars. ^***^P < 0.001, ^**^P < 0.01, ^*^P < 0.05. P-values are provided in Supplementary Table 3.

KA neurons were identified almost 100 years ago in over 200 vertebrates by Kolmer and Agduhr (hence the name “KA neurons”). These cells are GABAergic and have ipsilateral ascending axons. Notably, they are located near the central canal and their apical dendritic extensions extend microvilli and a motile cilium into the canal and contact cerebrospinal fluid (CSF) (e.g., Kolmer, 1921; Agduhr, 1922; Vigh et al., 1977; Barber et al., 1982; Dale et al., 1987; Bernhardt et al., 1992; Roberts et al., 1995; Stoeckel et al., 2003). This suggests that these neurons may modulate spinal cord functions in response to changes in CSF composition and/or flow. Consequently, more recently they also been called CSF-contacting neurons (CSF-cNs). CSF-cNs/KA neurons can be divided into distinct ventral and dorsal populations called KA″ or KA′ neurons, respectively (Figure 1; Park et al., 2004; Djenoune et al., 2014; Petracca et al., 2016). KA″ neurons are located in the most ventral part of the spinal cord and originate from the most ventral progenitor domain, the p3 domain, which also produces V3 ventral interneurons (Park et al., 2004; Schäfer et al., 2007; Djenoune et al., 2014; Petracca et al., 2016). In contrast, KA′ neurons are located slightly more dorsally and in zebrafish they originate from the progenitor domain that is located just above the p3 domain, the pMN domain, which also produces motoneurons (MNs) (Park et al., 2004; Djenoune et al., 2014). However, in mouse these more dorsal KA′ cells originate from both the pMN progenitor domain and the progenitor domain just above it, the p2 domain, which also generates V2 interneurons (Petracca et al., 2016). Excitingly, recent studies have started to elucidate the functions of these KA neurons/CSF-cNs and have discovered differences between them. In mouse, the two classes of cells have distinct electrophysiological properties: most KA′ cells have repetitive spiking whereas KA″ cells only fire once (Petracca et al., 2016). In zebrafish, KA′ and KA″ neurons express distinct neuropeptides, have slightly different axonal and dendritic morphologies and have both overlapping and distinct synaptic partners and functions in locomotor circuitry (Bohm et al., 2016; Hubbard et al., 2016; Djenoune et al., 2017). For example, KA′ neurons respond to lateral bending of the spinal cord and regulate both the duration and the frequency of slow locomotion while KA″ neurons respond to longitudinal contractions and regulate posture during fast locomotion (Bohm et al., 2016; Hubbard et al., 2016; Djenoune et al., 2017). However, despite these recent advances in understanding KA neuronal properties and functions we currently know very little about how these two cell types are specified.

V2b neurons (also called VeLDs in zebrafish) develop dorsal to KA neurons, from the p2 progenitor domain. Similar to KA neurons, they are GABAergic, and their axons are ipsilateral, but in contrast to KA neurons, V2b axons descend toward the caudal end of the spinal cord. V2b neurons also have important functions in locomotion circuitry. For example, V2b neurons prevent extensor and flexor muscles from contracting simultaneously, so enabling the alternating muscle contraction that is essential for walking (Al-Mosawie et al., 2007; Batista et al., 2008; Kimura et al., 2008; Joshi et al., 2009; Zhang et al., 2014; Britz et al., 2015). However, like KA neurons, we still do not fully understand how the development of V2b neurons is genetically regulated.

Zebrafish KA″, KA′, and V2b cells all express *tal1* (previously called *scl*), *gata3*, and *gata2a* [previously called *gata2*, (*gata2b* is not expressed in spinal cord, Lewis Lab unpublished data); (Batista et al., 2008; Kimura et al., 2008; Butko et al., 2015)]. *gata2a* and *gata3* encode C4 zinc-finger transcription factors and *tal1* encodes a basic helix-loop-helix transcription factor. All three of these transcription factors are also expressed in amniote V2b cells (Nardelli et al., 1999; Zhou et al., 2000; Karunaratne et al., 2002; Smith et al., 2002; Li et al., 2005; Muroyama et al., 2005; Al-Mosawie et al., 2007; Del Barrio et al., 2007; Peng et al., 2007) and Gata2 and Gata3 are expressed by amniote CSF-cN/KA neurons (Petracca et al., 2016; expression of Tal1 was not examined). In mouse, Gata2 is required for generation of correct numbers of both V2a and V2b cells (Nardelli et al., 1999; Zhou et al., 2000; Francius et al., 2015), but it is not clear whether the “missing” V2 cells die, fail to differentiate or transfate into a different cell type in these mouse *Gata2* mutants. In contrast, when Tal1 function is eliminated in the mouse CNS, V2b cells transfate into V2a cells (Muroyama et al., 2005; Joshi et al., 2009). However, in both of these mouse mutants, CSF-cNs/KA neurons were not analyzed.

In contrast, experiments in zebrafish have examined functions of Gata2a and Gata3 in KA cells but not V2b neurons. Interestingly, morpholino knock-down of Gata2a in zebrafish resulted in a loss of GABAergic and *gata3*- and *tal2*- expressing cells in the region of the spinal cord where KA″ cells normally form (the most ventral row of the spinal cord) but not the region where KA′ cells are located (the two rows dorsal to the most ventral row). In contrast, morpholino knock-down of Gata3 resulted in a loss of GABAergic and *tal2*-expressing cells in the KA′ but not the KA″ region, suggesting that even though these transcription factors are expressed by both KA′ and KA″ cells, they may be differentially required by these cells (Yang et al., 2010). However, similar to the mouse *Gata2* mutant analyses discussed above, these zebrafish experiments did not determine whether the “missing” cells die, transfate, or just lose their GABAergic phenotypes. In addition, morpholinos can sometimes cause non-specific off-target effects (Kok et al., 2015), so it is important to confirm these phenotypes with mutant analyses.

Tal1 function(s) in KA cells and Gata3 function(s) in V2b cells have not been examined in any vertebrate. While mouse *Gata3* mutants exist (e.g., Pandolfi et al., 1995; Pai et al., 2003; Craven et al., 2004; Zhang et al., 2004; Kurek et al., 2007), spinal interneurons have not been examined in these mutants. It is also not known whether any of these three genes act redundantly in spinal neurons.

To address all of these fundamental gaps in our knowledge, we performed detailed analyses of the spinal cord phenotypes of zebrafish *tal1, gata3*, and *gata2a* single and double mutants. We also examined *sox1* expression in the zebrafish spinal cord, demonstrating for the first time that *sox1a* and *sox1b* are expressed by both KA″ and KA′ neurons. These *sox1* experiments also revealed that, unlike in amniotes where Sox1 is expressed by a small subset of V2 cells called V2c cells, zebrafish *sox1* genes are expressed by at least most V2b neurons, suggesting that V2c cells may not exist in zebrafish. Interestingly, our *tal1, gata3*, and *gata2a* mutant analyses suggest that each of the transcription factors encoded by these genes is only required in some of the spinal neurons that co-express these genes. Gata2a is required in KA″ neurons to specify KA″ fates and repress V3 fates, but it is not required for correct development of KA′ neurons. In contrast neither Gata3 nor Tal1 are required for correct development of KA″ neurons, either singly or redundantly, but both of these transcription factors are required for KA′ neurons to differentiate from progenitor cells. Finally, we also demonstrate that all three of these transcription factors are required for later stages of V2b neuron development.

## Materials and methods

### Ethics statement

All zebrafish experiments in this research were carried out in accordance with the recommendations of, and were approved by, the Syracuse University IACUC committee.

### Zebrafish husbandry and fish lines

Zebrafish (*Danio rerio*) were maintained on a 14-h light/10-h dark cycle at 28.5°C. Embryos were obtained from natural paired and/or grouped spawnings of wild-type (WT) (AB, TL or AB/TL hybrid), *Tg(-8.1gata1:gata1-EGFP)* (Kobayashi et al., 2001), *gata2a*^*um27*^ (Zhu et al., 2011), *gata3*^*sa0234*^ (described here), or *tal1*^*t21384*^ (Bussmann et al., 2007) fish. Embryos were staged in hours post fertilization at 28.5°C (h) according to Kimmel et al. (1995).

The *Tg(-8.1gata1:gata1-EGFP)* (Kobayashi et al., 2001) transgenic line expresses EGFP in all KA (KA″ and KA′) neurons and some V2b neurons (Batista et al., 2008).

The *gata2a*^*um27*^ and *tal1*^*t21384*^ mutants have been previously described and are both presumed to be null alleles (Bussmann et al., 2007; Zhu et al., 2011). The *gata2a* mutation is a 10 bp deletion that creates a stop codon upstream of both zinc finger domains (Zhu et al., 2011). The *tal1* mutation is a nonsense mutation that produces a stop codon at amino acid 183, upstream of the C-terminus of the protein, including the entire bHLH domain (Bussmann et al., 2007). Therefore, in each case, even if a truncated protein is made, it should be unable to bind DNA.

The *gata3*^*sa0234*^ mutation was created using zinc finger nucleases by Huw Williams, Steve Harvey, and Ross Kettleborough in the Stemple Lab at the Wellcome Trust Sanger Centre. If translated, this mutant allele would encode a truncated protein with 13 aberrant amino acids after the Threonine at position 264, followed by a premature stop. As a result, only 8 amino acids of the first zinc finger domain would remain intact, and the second zinc finger would be completely lost (Supplementary Figure 1), strongly suggesting that this is a null allele. Consistent with this, our analyses of KA cell expression of *tal2* and *gad67* in *gata3* mutants are consistent with earlier morpholino knock-down analyses of *gata3* function in KA cell development (Yang et al., 2010). We also observed a one-to-one correspondence between mutant phenotypes and a homozygous mutant genotype, consistent with the phenotypes resulting from the loss of Gata3 function.

### Genotyping

DNA for genotyping was isolated from both anesthetized adults and fixed embryos via fin biopsy or head dissections, respectively. PCR and restriction enzyme digest assays or KASP assays designed by LGC Genomics LLC, using DNA extracted from head dissections, were used to identify fish carrying mutations. KASP assays use allele-specific PCR primers, which differentially bind fluorescent dyes that we quantified with a BioRad CFX96 real-time PCR machine to distinguish genotypes. The proprietary primers used are: Gata2_um27 (*gata2a* genotyping), Gata3_sa0234 (*gata3* genotyping), and Tal1_t21384 (*tal1* genotyping).

Heads of fixed embryos were dissected in 70% glycerol/30% phosphate-buffered saline (PBS) with insect pins. Embryo trunks were stored in 70% glycerol/30% PBS at 4°C for later analysis. DNA was extracted via the HotSHOT method (Truett et al., 2000) using 20 μL of 50 mM NaOH and 2 μL of 1M Tris-HCl (pH-7.5).

The *gata2a*^*um27*^ mutation results in a 10 bp deletion and was PCR genotyped using primers and protocol described in Zhu et al. (2011). The PCR produces a 98 bp product from the mutant allele and a 108 bp product from the WT allele. These products were separated and identified using a 2% Super Fine Resolution (SFR) agarose gel. Alternatively, a different PCR was performed using forward 5′TTTTCCGTGACCCTGTGTTC and reverse 5′ACTCACCAGTCTGCGCTTTG primers and reaction conditions of: 98°C for 60 s followed by 34 cycles of 94°C for 30 s, 61°C for 45 s, 72°C for 30 s and a final extension at 72°C for 5 min. This PCR reaction generates a product of 264 bp, which was digested using Msp1. Msp1 does not cut the WT PCR product, but cuts the mutant PCR product generating 164 and 100 bp fragments.

*tal1*^*t21384*^ mutants were identified by PCR using forward 5′TTTCATGCGCATATCCAAAA and reverse 5′GAAAATCCGTCGCACAACT primers and the following conditions: 98°C for 3 min followed by 34 cycles of 94°C for 30 s, 54°C for 45 s, 72°C for 30 s and a final extension at 72°C for 5 min. This PCR reaction generates a product of 180 bp. This was digested using the DdeI restriction enzyme, which does not cut the 180 bp WT PCR product but cuts the mutant PCR product to generate 160 and 20 bp fragments.

*gata3*^*sa0234*^ mutants were genotyped by PCR using the following primers: forward 5′GGTTGTGTAGTTGTGCTTGC and reverse 5′TTCTGTCCGTTCATCTTGTG and the following conditions: 98°C for 60 s followed by 34 cycles of 94°C for 30 s, 58°C for 45 s, 72°C for 30 s and a final extension at 72°C for 5 min. This generates a PCR product of 240 bp. This was digested using the Hinf1 restriction enzyme, which does not cut the mutant PCR product but cuts the WT PCR product to generate 159 and 81 bp fragments. These products were separated and identified using a 2.5% Super Fine Resolution (SFR) agarose gel.

### *In situ* hybridization and immunohistochemistry

Embryos were fixed in 4% paraformaldehyde and single *in situ* hybridization or fluorescent *in situ* hybridization plus immunohistochemistry experiments were performed as previously described (Concordet et al., 1996; Batista et al., 2008). Embryos older than 24 h were often incubated in 0.003% 1-phenyl-2-thiourea (PTU) to prevent pigment formation. For fluorescent *in situ* hybridization + immunohistochemistry, after detection of the *in situ* hybridization reaction using TSA Kit #5, with HRP, Goat anti-mouse IgG and Alexa Fluor 594 Tyramide (ThermoFisher Scientific, T20915), embryos were washed 8 × 15 min in PBST (PBS with 0.1% Tween-20) and incubated in Image-iT FX Signal Enhancer (ThermoFisher Scientific, I36933) for 30 min at room temperature. Immunohistochemistry was performed using the following primary antibodies: chicken polyclonal anti-GFP primary antibody (Abcam, Ab13970, 1:500), mouse anti-Nkx6.1 (F55A12, Develpmental Studies Hybridoma Bank, Iowa, 1:500), rabbit anti-phospho-Histone H3 (Ser10; Millipore #06-570; 1:500), rabbit anti-activated Caspase-3 (Fisher Scientific/BD, BDB559565, 1:500), a mixture of rat anti-Islet-1 and rat anti-Islet-2 (Developmental Studies Hybridoma Bank, Iowa antibodies 39.4D5 and 40.2D6 were mixed 1:1 and used at a final concentration of 1:300). The secondary antibodies used were: goat anti-rabbit Alexa Fluor 568 (ThermoFisher Scientific, A-11036, 1:1,000), and a goat anti-chicken IgY (H+L), Alexa Fluor 488 secondary antibody (ThermoFisher Scientific, A-11039, 1:1,000) and goat anti-mouse Alexa Fluor 488 (ThermoFisher Scientific, A-11029, 1:1,000). Both double *in situ* hybridization and immunohistochemistry plus *in situ* hybridization double labeling experiments were performed as previously reported (Batista et al., 2008). Immunohistochemistry for GFP and pH3 was performed as described in Juárez-Morales et al. (2016) and immunohistochemistry for Islet1/2 was performed as described in Lewis and Eisen (2004). Cross-sections were cut by hand using a razor blade mounted in a 12 cm blade holder (World Precision Instruments, Cat. # 14134). In cases where expression of a particular gene is lost in a specific region of the spinal cord, we checked for low levels of expression of that gene by substantially over-staining embryos.

To determine neurotransmitter phenotypes, we used *in situ* probes of genes that function as transporters of neurotransmitters or that synthesize specific neurotransmitters as these are some of the most specific molecular markers of these cell fates (Higashijima et al., 2004a,b and references therein). A mixture of probes to *slc17a6a* and *slc17a6b* (previously called *vglut*), which encode glutamate transporters, was used to label glutamatergic neurons (Higashijima et al., 2004a,b). GABAergic neurons were labeled by a mixture of probes to *gad1b* and *gad2* genes (probes previously called *gad67a, gad67b*, and *gad65*) (Higashijima et al., 2004a,b). The *gad1b* and *gad2* genes encode for glutamic acid decarboxylases, which are necessary for the synthesis of GABA from glutamate. The *sox1a* and *sox1b* probes were synthesized from plasmids obtained from Dr. Uwe Strähle (Karlsruhe Institute of Technology, Germany) from the library described by Armant et al. (2013). *gata2a, gata3, sst1.1*, and *urp1* probe templates were PCR amplified from 27 h WT zebrafish cDNA. The PCR primers used are provided in Supplementary Table 1. cDNA was prepared as described previously (England et al., 2017). In all cases, reverse primers contained the T3 RNA polymerase promoter binding site used to synthesize the antisense RNA probe. PCR conditions were: 94°C for 3 min followed by 35 cycles of 94°C for 30 s, 56.5°C for 30 s, 72°C for 1.5 min and a final extension step of 72°C for 10 min. All other probes were synthesized from plasmids that have previously been reported. For details and corresponding references please see Supplementary Table 2.

To assess expression of Caspase3, rabbit Anti-Activated Caspase-3 (Fisher Scientific/BD, BDB559565, 1:500) was used as described previously (Hilinski et al., 2016). Twenty-four hours embryos were fixed in 4% PFA at 4°C overnight, washed 3 times in PBST, permeabilized with acetone for 20 min at −20°C and then washed 3 × 5 min with PBS. Embryos were then blocked (Blocking Solution: Triton 0.5%, BSA 2%, DMSO 10%, Goat Serum 2%, and PBS) for 2 h at room temperature and incubated overnight with an Anti-Activated Caspase-3 primary antibody diluted 1:500 in blocking solution at 4°C. The next day, embryos were washed for 8 × 15 min in PBTX (PBS with 0.5%, Triton and 5% DMSO) and incubated with goat Anti-Rabbit Alexa 488 (1:500) in PBTX +2% BSA for 4 h at room temperature. Embryos were washed 8 × 15 min in PBTX and mounted in 2% DABCO solution for imaging and analysis.

### Imaging

Embryos were mounted in 70% glycerol, 30% PBS, and differential interference contrast (DIC) pictures were taken using an AxioCam MRc5 camera mounted on a Zeiss Axio Imager M1 compound microscope. A Zeiss LSM 710 confocal microscope was used to image fluorescent *in situ* and fluorescent immunohistochemistry experiments. All confocal images were processed using Image J software (Abràmoff et al., 2004), in which case appropriate numbers of focal planes were merged using maximum intensity projections. For some NBT-BCIP ISH experiments, multiple planes were merged in Image J using minimum intensity projections in order to show labeled cells at different medial lateral positions in the spinal cord. All images were processed for brightness-contrast and color balance using Adobe Photoshop software (Adobe, Inc.). Images of control and mutant embryos from the same experiment were processed identically. Figures were assembled using Adobe Illustrator (Adobe, Inc.).

### Counting cells

Embryos from single and double mutant crosses were counted blind to genotype. The row immediately dorsal to the notochord is denoted as row 1 and rows dorsal to this are assigned in ascending order (e.g., Figures 1A,D). In all cases, cell counts are for both sides of a five-somite length of the spinal cord adjacent to somites 6–10. Embryos were mounted laterally with the somite boundaries on each side of the embryo exactly aligned and the apex of the somite over the middle of the notochord. This ensures that the spinal cord is straight along its dorsal-ventral axis and that cells in the same dorsal/ventral position on opposite sides of the spinal cord will be directly above and below each other. In some cases the medial-lateral location of labeled cells was also determined to distinguish between V2b and KA cells. KA cells are medial and V2b cells are more lateral. Based on our analyses of several different genes expressed by KA and/or V2b cells, we assigned medial cells in row 1 expressing these genes as KA″ cells, medial cells in rows 2 and 3 expressing these genes as KA′ cells and lateral cells in rows 3 and above expressing these genes as V2b cells (e.g., Figures 1A–E). However, note that *gad* genes are also expressed in more dorsal spinal cells (e.g., see Batista and Lewis, 2008). Labeled cells in embryos analyzed by DIC were counted while examining embryos on a Zeiss Axio Imager M1 compound microscope. We identified somites 6–10 in each embryo and counted the number of labeled cells in that stretch of the spinal cord. We adjusted the focal plane as we examined the embryo to count cells at all medial/lateral positions (both sides of the spinal cord; also see Batista and Lewis, 2008; Batista et al., 2008; England et al., 2011; Hilinski et al., 2016; Juárez-Morales et al., 2016). Cell counts for fluorescently-labeled cells were performed by analyzing all focal planes in a confocal stack of the appropriate region of the spinal cord. For Islet 1/2-positive cells, cells in the two most dorsal rows, which correspond to Rohon-Beard neurons, were not counted. Only ventral cells that correspond to motoneurons were counted. These also have smaller nuclei than the more dorsal Rohon Beard cells. For pH3-positive cells, cell rows were assigned based on average cell diameters. In all cases, values are reported as the mean ± the Standard Error of the Mean (SEM). To determine whether differences in values are statistically significant, data were first analyzed for normality using the Shapiro–Wilk test. Data sets with non-normal distributions were subsequently analyzed using the Wilcoxon (Mann–Whitney) test. For data sets with normal distributions, the F test for equal variances was performed, prior to conducting either a type 2 (for equal variances) or type 3 (for non-equal variances) student's *t*-test. P values generated by Wilcoxon, type 2 student's *t*-test and type 3 student's *t*-test are indicated by the symbols ^∧^, ^+^, and ^§^, respectively, in Supplementary Tables 3, 6. *n*-values for each experiment are provided in the figure legends.

## Results

### Tal1 is required for expression of *gata3, gata2a*, and *tal2* in the KA′ spinal region

We have previously shown that KA″, KA′, and V2b spinal neurons express *tal1* (Batista et al., 2008; see also Figures 1B,C,E), but the function(s) of Tal1 in these cells has not been investigated. Therefore, as a first step to determining if Tal1 is required for correct development of these cells, we examined other markers of KA and V2b cells in a zebrafish *tal1* mutant (Bussmann et al., 2007).

**Figure 2:**
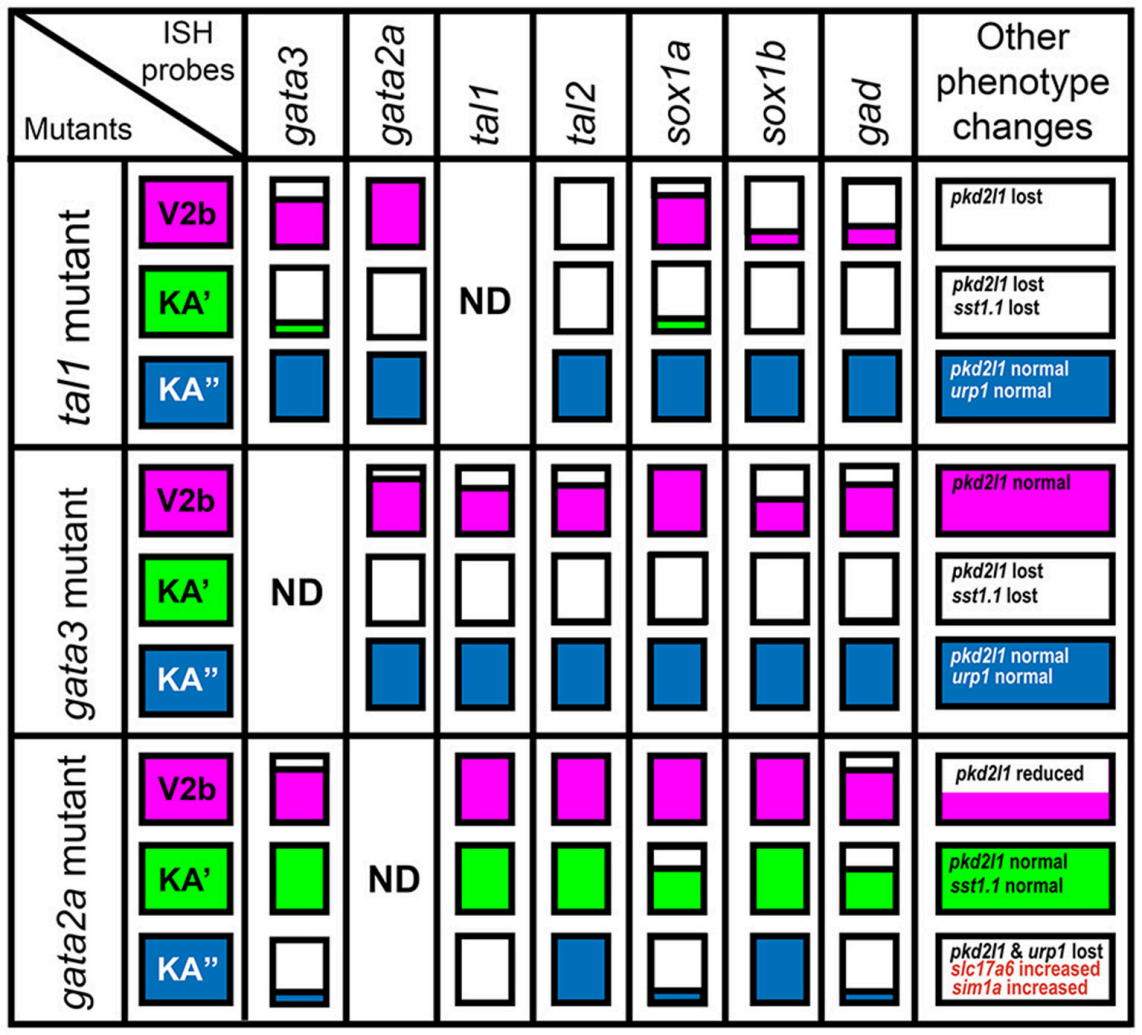
Gene expression phenotypes in *gata2a, gata3*, and *tal1* mutants. Summary schematic representation of gene expression phenotypes in *gata2a, gata3*, and *tal1* mutants. Phenotypes are reported for cells in the spinal cord regions where KA″, KA′, and V2b cells normally form. The KA″ region (blue) corresponds to row 1 cells, the KA′ region (green) corresponds to all row 2 plus medial row 3 cells and the V2b region corresponds to lateral row 3 cells plus all row 4 and above cells. White boxes indicate that no cells in that spinal cord region express the gene indicated at top of column, in the mutant indicated in the most left hand column. A box filled with color indicates that there is no change in the number of cells expressing that particular gene in that region. A box partially filled with color indicates a partial reduction in the number of cells expressing the gene. The amount of the box that is white is approximately proportional to the degree of reduction. Information on additional genes that are not expressed in all cell types is provided in the final column.

In wild type (WT) embryos, *gata3, gata2a*, and *tal2* are all expressed by KA″ and KA′ neurons (Pinheiro et al., 2004; see also Figures 1F,I,L; Batista et al., 2008). In addition, *gata3* and *gata2a* are expressed by V2b neurons and *tal2* is expressed by a subset of V2b neurons (Figures 1F,I,L; Supplementary Figure 2; also see Batista et al., 2008; Yang et al., 2010). In *tal1* mutants, we found no change in the number of cells expressing any of these genes in row 1 (Figures 1F–N, 2; Supplementary Table 3). In WT embryos, row 1 cells that express these genes are KA″ neurons (Batista et al., 2008; see also Figure 1 and section Materials and Methods). We also found no change in the number of cells expressing *gata3* or *gata2a* in row 4 or above (Figures 1F–K, 2; Supplementary Table 3), although *tal2* expression was lost in this region (Figures 1L–N, 2; Supplementary Table 3). In WT embryos, the cells that express these genes in this spinal cord region are V2b neurons (Batista et al., 2008; see also Figure 1 and section Materials and Methods). In contrast, there was a dramatic reduction in the number of cells expressing each of these three genes in rows 2 and 3 in *tal1* mutants (Figures 1F–N, 2; Supplementary Table 3). In WT embryos, the row 2 cells that express these genes are KA′ cells but row 3 cells that express these genes might be KA′ or V2b cells (Batista et al., 2008; see also Figures 1A–D and section Materials and Methods). KA′ and V2b cells can be distinguished by their locations and soma-morphologies. KA′ cells are medial and have more circular soma, whereas V2b cells are lateral and have pear-shaped soma (Figures 1A–C). All of the remaining row 3 *gata3*- and *gata2a*-expressing cells in the *tal1* mutants were pear shaped and located laterally, consistent with them being V2b cells (Figure 1C; Supplementary Table 4). The most likely explanation of these results is that either KA′ cells are lost in *tal1* mutants or that *tal1* is required for expression of *gata3, gata2a* and *tal2* in KA′ cells. In contrast, these data suggest that *tal1* is not required for expression of *gata3, gata2a* or *tal2* in KA″ cells or *gata3* or *gata2a* in V2b cells, although it is required for expression of *tal2* in V2b cells.

### Gata3 is required for expression of *gata2a, tal1*, and *tal2* in the KA′ region whereas gata2a is necessary for the expression of *gata3* and *tal1* in the KA″ region of the spinal cord

Previous morpholino experiments suggested that Gata3 and Gata2a may have distinct functions in KA′ and KA″ cells, respectively (Yang et al., 2010). However, morpholinos can cause non-specific defects (Kok et al., 2015) and the function of these genes in zebrafish V2b neurons was unknown. Therefore, to determine if Gata3 and/or Gata2a are required for correct development of zebrafish V2b or KA cells, we examined markers of these cell types in a *gata2a* mutant (Zhu et al., 2011) and a newly-generated *gata3* mutant (see section Materials and Methods).

**Figure 3:**
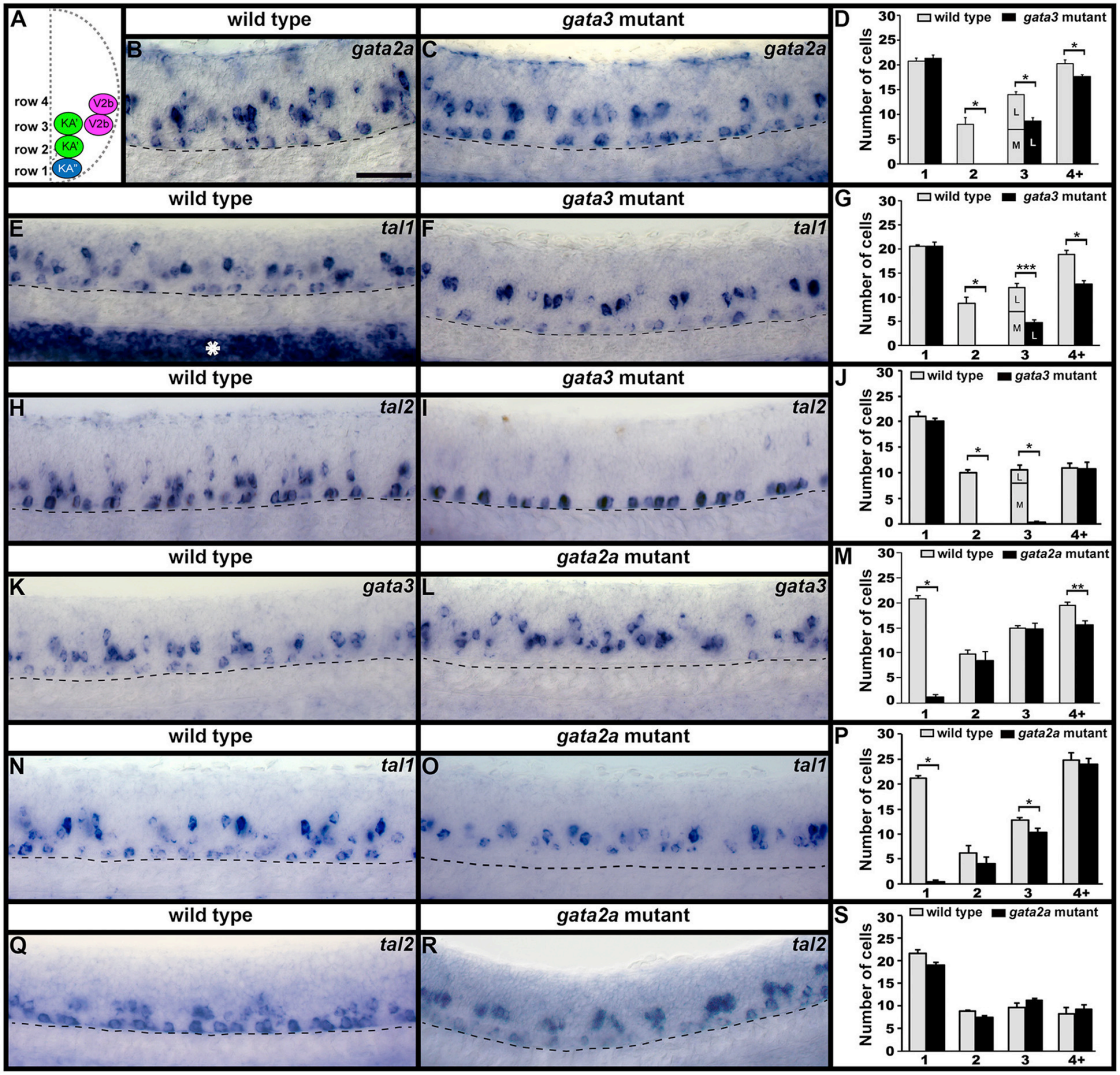
*gata2a, gata3, tal1*, and *tal2* expression in KA″, KA′, and V2b neurons is differentially regulated by *gata3* and *gata2a*. **(A)** Cross-sectional schematic indicating positions of KA″, KA′, and V2b neurons. Lateral views of *gata2a* **(B,C)**, *tal1* **(E,F,N,O)**, *tal2* **(H,I,Q,R)**, and *gata3* **(K,L)** expression in 24 h WT siblings **(B,E,H,J,N,Q)** and *gata3* **(C,F,I)** and *gata2a* **(L,O,R)** mutants. Dorsal, top; in lateral views, anterior, left. White asterisk in **(E)** indicates blood expression of *tal1*. Dashed lines indicate ventral limit of spinal cord. Scale bar = 50 microns. Mean number of cells expressing each gene in each D/V row of spinal cord region adjacent to somites 6–10 **(D,G,J,M,P,S)**. For *gata3* mutant results **(D,G,J)** the approximate proportions of medial and lateral row 3 cells are indicated by horizontal lines separating the number of medially-located cells (bottom and indicated with an “M”) from the number of laterally-located cells (top and indicated with a “L”). All of the remaining *gata2a*- and *tal1*-expressing cells in row 3 of *gata3* mutants were located laterally, suggesting that they are V2b cells and that no KA′ cells express these genes in *gata3* mutants. More dorsal (row 4 and above) cells expressing *tal2* in *gata3* mutants **(I)** are more weakly stained than the strongly stained KA″ cells and are in a different focal plane. All counts are an average of at least 4 embryos. Error bars indicate SEM. Statistically significant comparisons are indicated with brackets and stars. ^***^P < 0.001, ^**^P < 0.01, ^*^P < 0.05. For *P*-values see Supplementary Table 3.

In *gata3* mutants, we found no change in the number of cells expressing *gata2a, tal1*, or *tal2* in row 1 (KA″ region; Figures 2, 3B–J; Supplementary Table 3). However, there was a complete loss of expression of all three genes in row 2 and a statistically significant reduction in the number of cells expressing each gene in row 3 (KA′ region; Figures 2, 3B–J; Supplementary Table 3). As with *tal1* mutants, all of the remaining labeled row 3 cells were located laterally, suggesting that none of these cells are KA′ neurons (Figures 3D,G,J; Supplementary Table 5). However, in contrast to *tal1* mutants, in *gata3* mutants there was also a statistically significant decrease in the number of cells expressing *gata2a* and *tal1* in row 4 and above, suggesting that there may also be a reduction in the number of V2b cells expressing these genes (Figures 2, 3B–G; Supplementary Table 3). Together, these results suggest that *gata3* is not required for expression of *gata2a, tal1*, or *tal2* in KA″ cells or most V2b cells. However, it is probably required for KA′ cell differentiation, KA′ cell survival or expression of *gata2a, tal2*, and *tal1* in KA′ cells.

In contrast to both *gata3* and *tal1* mutants, in *gata2a* mutants there was no statistically significant change in the number of cells expressing *gata3* or *tal2* in rows 2 or 3 or the number of cells expressing *tal1* in row 2 (KA′ region; Figures 2, 3K–S; Supplementary Table 3). However, we very rarely observed cells expressing *gata3* or *tal1* in row 1 (KA″ region; Figures 2, 3K–P; Supplementary Table 3). The number of cells expressing *tal2* in row 1 was unchanged, although the expression of *tal2* might be slightly reduced compared to WT embryos (Figures 2, 3Q–S; Supplementary Table 3). There was no statistically significant change in the number of cells expressing *tal1* or *tal2* in row 4 and above. However, there was a small, but statistically significant, decrease in the number of cells expressing *gata3* in this spinal cord location (V2b region; Figures 2, 3M; Supplementary Table 3). Taken together, these data suggest that *gata2a* is not required for expression of *gata3* or *tal2* in KA′ cells or for expression of *tal1* in at least most KA′ cells. However, our data suggest that *gata2a* is required for correct specification and/or development of KA″ cells and a small number of V2b cells. We do not think that either of these cell types are dying in *gata2a* mutants as we see normal numbers of *tal2*-expressing cells in row 1 and normal numbers of *tal1*- and *tal2*-expressing cells in row 4 and above.

### *sox1a/b* are expressed by KA″, KA′, and V2b neurons in zebrafish

**Figure 4:**
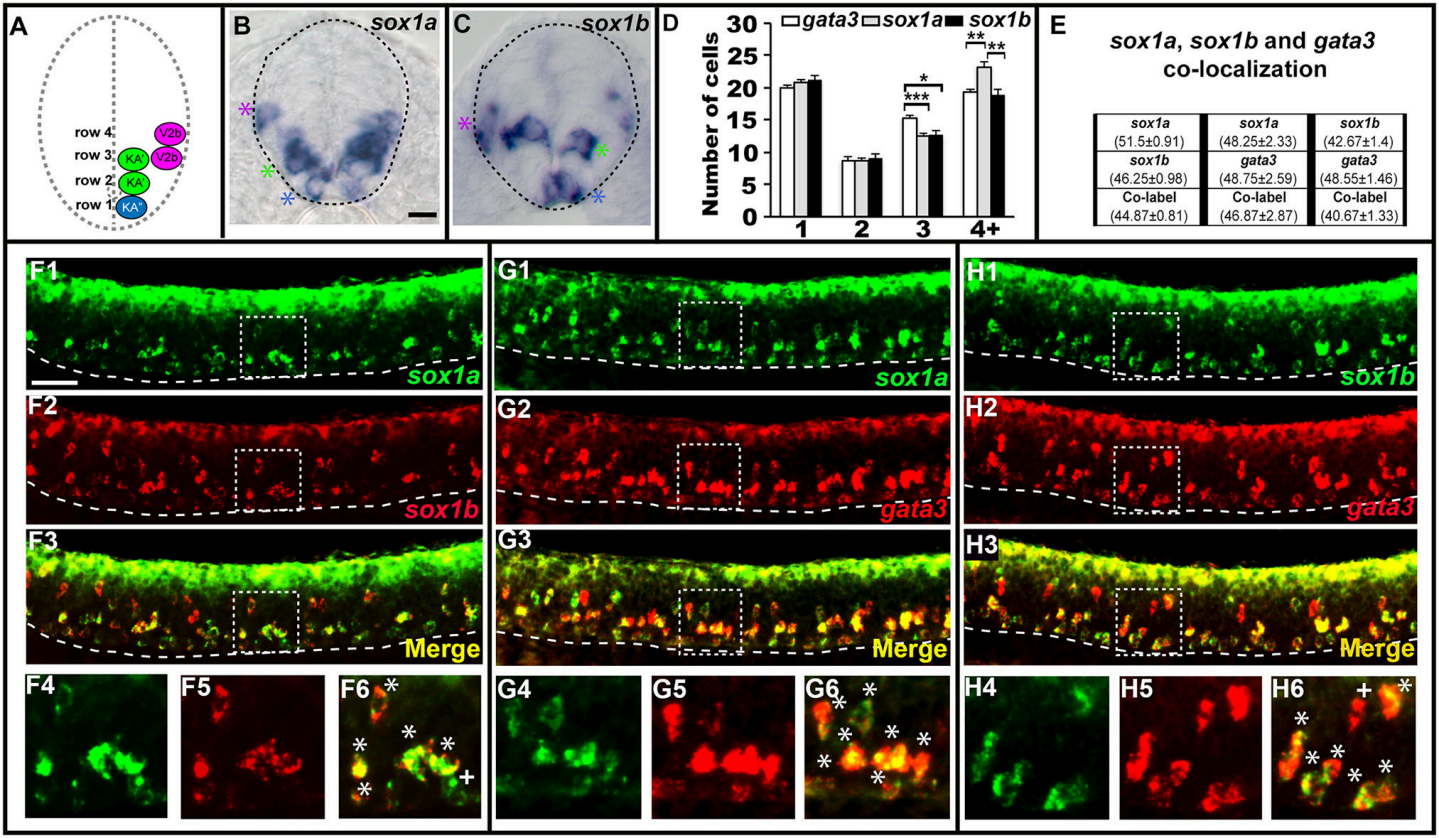
*sox1a* and *sox1b* are co-expressed in KA″, KA′, and V2b cells. Cross-sectional **(A–C)** and lateral **(F–H)** views of 24 h WT zebrafish embryos. Dorsal, top; in lateral views, anterior is left. Dashed lines indicate spinal cord boundary **(A–C)** or ventral limit of the spinal cord **(F–H)**. **(A)** Schematic indicating positions of KA″, KA′, and V2b neurons. **(B)** *sox1a* and **(C)** *sox1b* expression in KA″ (blue asterisks), KA′ (green asterisks), and V2b (magenta asterisk) cells. **(D)** Mean number of cells expressing *gata3, sox1a*, or *sox1b* in each D/V row of spinal cord region adjacent to somites 6–10. All counts are an average of at least 5 embryos. Error bars indicate SEM. Statistically significant (*P* < 0.05) comparisons are indicated with square brackets and stars. ^***^P < 0.001, ^**^P < 0.01, ^*^P < 0.05. There are no statistically significant differences between the numbers of cells expressing each of these genes in rows 1 and 2. While there are differences in rows 3 and 4, these are small and may reflect slight differences in timing or levels of expression and/or “noise” between different experiments as each gene was analyzed in different embryos. For *P*-values of all comparisons see Supplementary Table 3. **(E)** Quantification of single and double-labeled cells from double fluorescent *in situ* hybridization experiments **(F–H)**. Each column indicates the mean number of cells ±SEM expressing each gene and co-expressing the two genes. Double fluorescent *in situ* hybridization co-expression experiments showing expression of *sox1a* (green) and *sox1b* (red) **(F)**, *sox1a* (green), and *gata3* (red) **(G)**, *sox1b* (green) and *gata3* (red) **(H)**. Bottom row panels are single confocal plane magnified views of the dashed box regions in panels above. White asterisks in **(F6,G6,H6)** indicate cells co-expressing both genes whereas white crosses indicate single-labeled cells. Dorsal labeling in **(F1–H3)** is background accumulated from several confocal planes. As labeling is often weaker in double *in situ* hybridization experiments, cells that are apparently single-labeled may co-express the other gene at levels undetected in these experiments. Consistent with this, slightly fewer cells are labeled with each *in situ* hybridization probe in the double-labeling experiments than in the single *in situ* hybridization experiments. In addition, occasional single-labeled cells may result from one of the genes being expressed slightly earlier than the other. Scale bars **(B)** = 10 microns **(B,C)**; **(F1)** = 50 microns **(F1–H3)**.

In most of the cases described above, when there is a mutant phenotype in the V2b region, there is only a partial reduction in the number of cells expressing the gene in question. The only exception is *tal2* expression in *tal1* mutants (Figure 2) and *tal2* is itself only expressed in a subset of V2b cells. In mice, a small subset of V2b cells becomes a distinct population of cells called V2c cells (Panayi et al., 2010). Therefore, we hypothesized that the affected cells in our mutants might be V2c cells. However, it had not yet been established if V2c cells exist in zebrafish. In mouse, V2c cells are the only V2 cells that express Sox1 (Panayi et al., 2010). Therefore, we examined spinal cord expression of *sox1a* and *sox1b*, the zebrafish orthologs of mouse *Sox1* (Okuda et al., 2006). Interestingly, we found that both *sox1a* and *sox1b* are expressed in similar numbers of cells not just in the V2 spinal cord region but also in the KA″ and KA′ regions (Figure 4), suggesting that these two genes might be co-expressed by both V2 and KA neurons. Co-localization experiments between *sox1a, sox1b* and *gata3* confirmed that *sox1a* and *sox1b* are co-expressed in all KA″ and KA′ neurons and, unlike in mouse, both of these *sox1* genes are expressed in at least most, and probably all, V2b neurons (Figure 4). We do observe occasional cells that express just one of these genes, but this probably reflects slight differences in the timing of gene expression and/or low levels of expression that are hard to detect in these double labeling experiments.

### Tal1, gata3, and gata2a are required for normal expression of *sox1a/b* in KA and V2b regions

**Figure 5:**
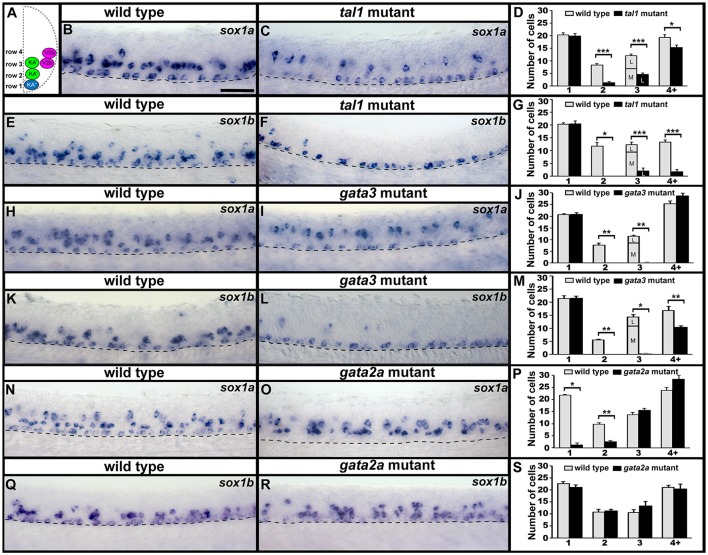
Expression of *sox1a* and *sox1b* in *tal1, gata3*, and *gata2a* mutants. **(A)** Cross-sectional schematic indicating positions of KA″, KA′, and V2b neurons. Lateral views of *sox1a* and *sox1b* expression in 24 h WT sibling **(B,E,H,K,N,Q)** and *tal1* **(C,F)**, g*ata3* **(I,L)**, and *gata2a* **(O,R)** mutants. Dorsal, top; anterior, left. Dashed lines indicate ventral limit of spinal cord. Scale bar = 50 microns. Mean number of cells expressing *sox1a* **(D,J,P)** or *sox1b* **(G,M,S)** in each D/V row of spinal cord region adjacent to somites 6–10 in WT and mutant embryos. For *tal1* and *gata3* mutant results **(D,G,J,M)** the approximate proportions of medial and lateral row 3 cells are indicated by horizontal lines separating the number of medially-located cells (bottom and indicated with an “M”) from the number of laterally-located cells (top and indicated with a “L”). All of the remaining row 3 cells in these mutants are lateral, consistent with them being V2b cells. All counts are an average of at least 4 embryos. Error bars indicate SEM. Statistically significant comparisons are indicated with brackets and stars. ^***^P < 0.001, ^**^P < 0.01, ^*^P < 0.05. For *P*-values see Supplementary Table 3.

As *sox1a* and *sox1b* are expressed in V2b, KA″, and KA′ cells we examined the expression of these genes in *tal1, gata3* and *gata2a* mutants. In *tal1* mutants, there was no effect on the number of row 1 cells expressing *sox1a* or *sox1b*, but there was a dramatic reduction in the number of cells expressing these genes in rows 2 and 3 (KA′ region; Figures 2, 5B–G; Supplementary Tables 3, 4). Interestingly there was also a severe reduction in the number of row 4 and above cells expressing *sox1b* but only a slight reduction in the number of these cells expressing *sox1a* (V2b region; Figures 2, 5B–G; Supplementary Table 3). In *gata3* mutants, there was also no effect on the number of row 1 cells expressing *sox1a* or *sox1b*, but there was a complete loss of *sox1a* and *sox1b* expression in rows 2 and 3 (KA′ region; Figures 2, 5H–M; Supplementary Tables 3, 5). There was no effect on the number of row 4 and above cells expressing *sox1a* but there was a slight reduction in the number of these cells expressing *sox1b* (V2b region; Figures 2, 5H–M; Supplementary Table 3). In contrast, in *gata2a* mutants there was no change in the number of cells expressing *sox1b*, but there was a reduction in the number of cells expressing *sox1a* in rows 1 and 2 (Figures 2, 5N–S; Supplementary Table 3).

Taken together, our results so far reveal that in *gata3* and *tal1* mutants, expression of all of the genes that we have examined is lost in the KA′ region (row 2 and medial row 3 cells), whereas in *gata2a* mutants expression of most of the genes that we have examined is lost in the KA″ region (row 1). There are several possible explanations for these phenotypes. We think it is unlikely that KA or V2b cells are mis-localized in any of these mutants, as we didn't observe an increase in the number of cells expressing any of these genes in any spinal cord region. Our data also suggest that cells that would normally develop as KA″ neurons are not dying in *gata2a* mutants as normal numbers of *sox1b*-and *tal2*-expressing cells are present in row 1 (Figures 3Q–S, 5Q–S). However, KA″ neurons could be mis-specified as a different cell type or just not expressing *sox1a, gata3*, and *tal1*. In *gata3* and *tal1* mutants, KA′ neurons could be dying, mis-specified as a different cell type or just not expressing the genes that we have examined.

### KA′ cells do not die or transfate into motoneurons or V2a cells in *tal1* or *gata3* mutants

**Figure 6:**
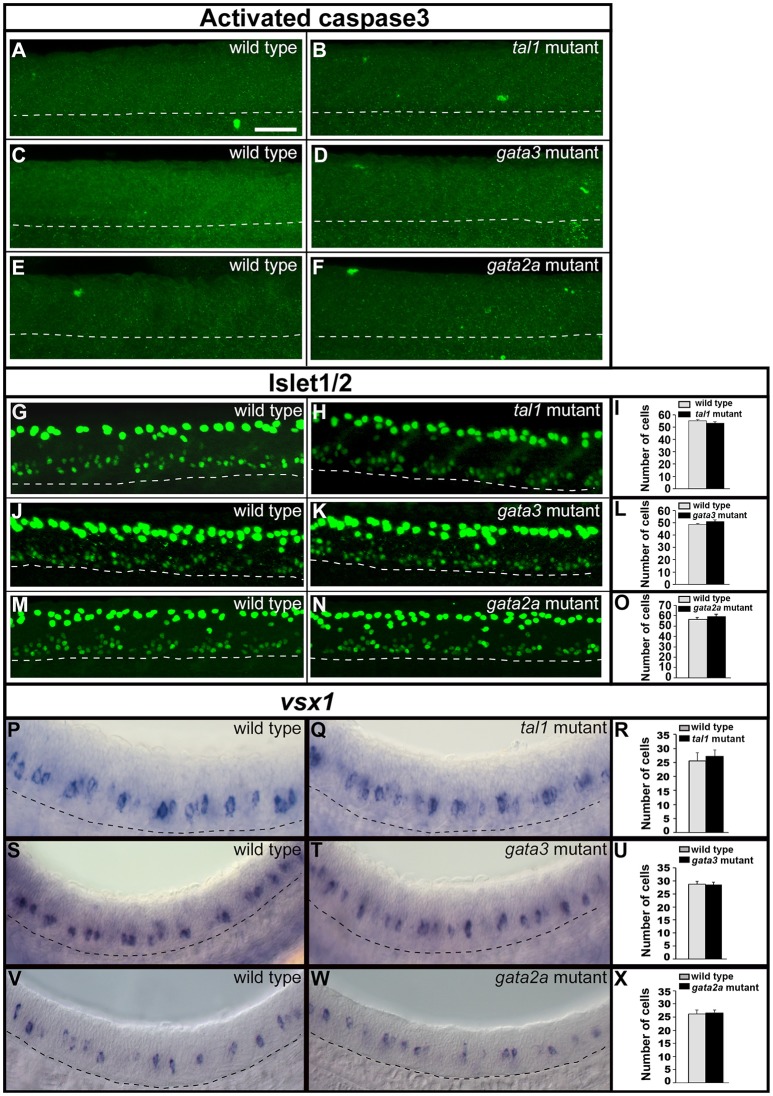
Activated caspase3 and Islet1/2 immunohistochemistry and expression of *vsx1* in wild type and mutant embryos. Lateral views of 24 h WT sibling and mutant embryos as indicated. Dashed lines mark ventral limit of spinal cord. Dorsal, top; anterior, left. Scale bar = 50 microns. **(A–F)** Immunohistochemistry for activated Caspase-3. Very few labeled cells are seen in WT or mutant embryos (0–3 cells in a 5 somite-length of spinal cord). **(G,H,J,K,M,N)** Immunohistochemistry for Islet-1 and Islet-2. The smaller labeled nuclei in the ventral spinal cord correspond to motoneurons. The larger more dorsal nuclei belong to Rohon-Beard cells. Mean numbers of Islet1/2 expressing motoneurons in WT and mutant embryos (**I,L,O**; see Supplementary Table 6 for *P*-values). Only ventral cells that correspond to motoneurons were counted. These also have smaller nuclei than more dorsally-located Rohon Beard cells. **(P,Q,S,T,V,W)** *vsx1* expression in V2a neurons. Mean numbers of *vsx1*-expressing V2a neurons in WT and mutant embryos (**R,U,X**, see Supplementary Table 6 for *P*-values). None of the comparisons contained in this figure revealed statistically significant differences.

To test whether KA and/or V2 neurons were dying in any of these mutants we used an activated caspase-3 antibody, as in previous zebrafish studies (Sorrells et al., 2013; Hilinski et al., 2016). However, we did not detect any increase in the number of labeled cells in *tal1, gata3*, or *gata2a* mutants compared to their WT siblings (Figures 6A–F).

To investigate whether KA or V2b neurons might be mis-specified as a different cell type in these mutants, we examined markers of motoneurons and V2a neurons, as these cell types are also present in KA and V2 regions in WT embryos at these stages. Motoneurons express Islet1/2 (Park et al., 2004) and V2a cells express *vsx1* (Kimura et al., 2006, 2008; Batista et al., 2008). However, there was no change in the number of cells expressing either of these markers in any of the three mutants (Figures 6G–X; Supplementary Table 6). This suggests that no cells in these mutants are mis-specified as motoneurons or V2a neurons.

### Tal1, gata3, and gata2a are required for gabaergic and peptidergic phenotypes of specific subsets of KA and V2b cells

As the precise functions of Tal1, Gata3 and Gata2a in KA and V2b development were still unclear, we next examined later functional properties of these cells, such as neurotransmitter and neuropeptide phenotypes, axonal projections and presence of a crucial channel protein. Our aims for these experiments were two-fold. First, we wanted to establish if any of the cells that have lost *sox1a, sox1b, gata2a, gata3, tal1*, and/or *tal2* expression still develop into functional KA or V2b neurons with appropriate morphological and/or physiological characteristics. Second, as the genes that we had examined so far are all expressed relatively early during KA and V2b development, we wanted to test if any of the cell types that had normal expression of these genes developed abnormal phenotypes later in development.

**Figure 7:**
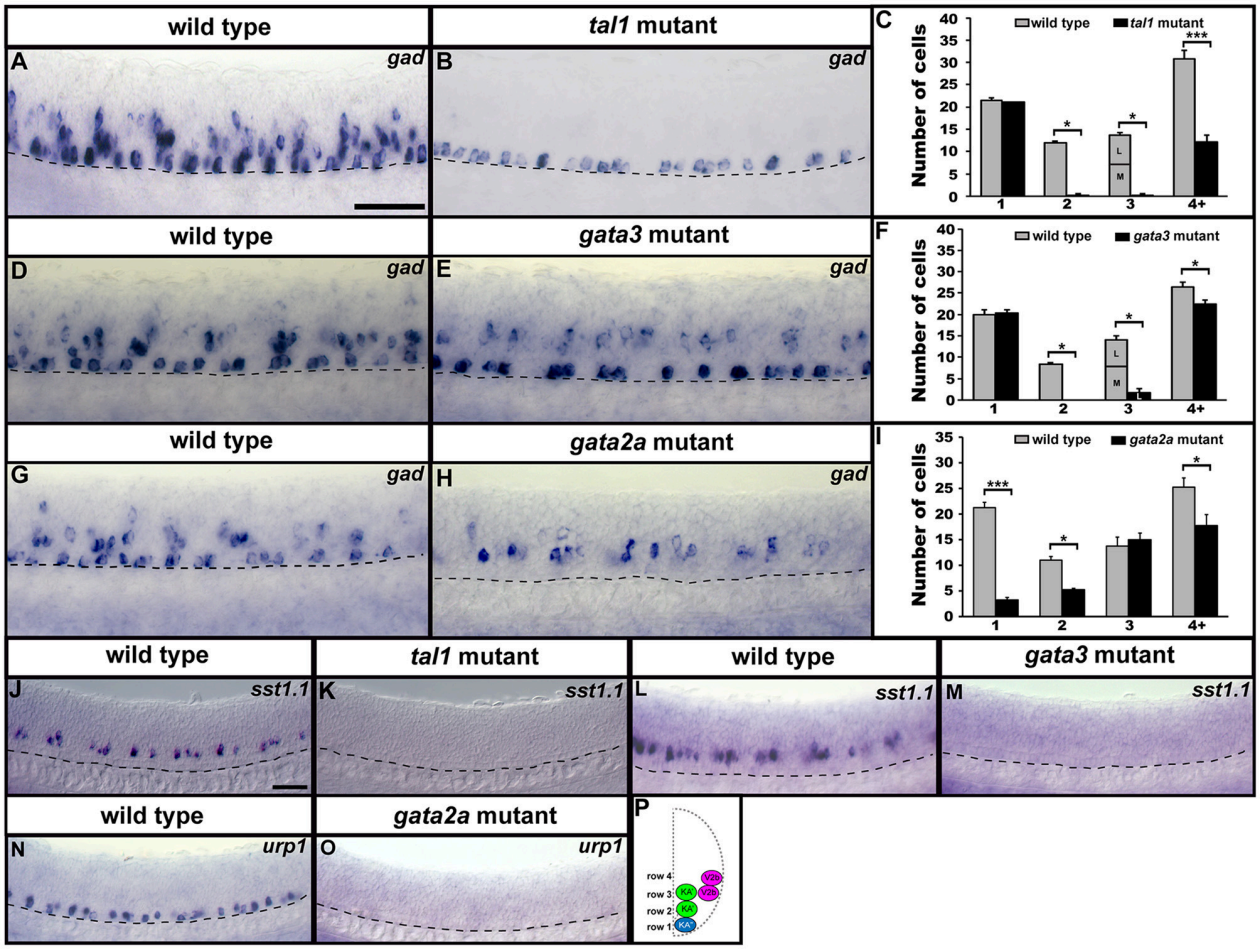
Neurotransmitter and neuropeptide phenotypes in *tal1, gata3*, and *gata2a* mutants. Lateral views of *gad, sst1.1*, and *urp1* expression in 24 h WT sibling **(A,D,G,J,L,N)** and *tal1* **(B,K)**, *gata3* **(E,M)**, and *gata2a* **(H,O)** mutants. Dorsal, top; anterior, left. Cross-sectional schematic indicating positions of KA″, KA′, and V2b neurons **(P)**. Scale bars **(A)** = 50 microns **(A,B,D,E,G,H)**; **(J)** = 50 microns **(J–O)**. Dashed lines indicate ventral limit of spinal cord. Mean number of cells expressing *gad* in each D/V row of spinal cord region adjacent to somites 6–10 in WT and mutant embryos **(C,F,I)**. For *tal1* and *gata3* mutant results **(C,F)** the approximate proportions of medial and lateral row 3 cells are indicated by horizontal lines separating the number of medially-located cells (bottom and indicated with an “M”) from the number of laterally-located cells (top and indicated with a “L”). All of the remaining row 3 cells in these mutants are lateral, consistent with them being V2b cells. More dorsal (row 4 and above) cells expressing *gad* in *tal1* mutants **(B)** are only weakly stained and are in a different focal plane to the strongly stained KA″ cells, so they are harder to see. All counts are an average of at least 4 embryos. Error bars indicate SEM. Statistically significant comparisons are indicated with brackets and stars. ^***^P < 0.001, ^*^P < 0.05. For *P*-values see Supplementary Table 3.

KA″, KA′, and V2b neurons are all GABAergic (Bernhardt et al., 1992; Batista et al., 2008). Therefore, we examined expression of *gad1b* and *gad2* (referred to here as *gad*), which are specifically expressed by GABAergic cells (see section Materials and Methods; *gad1b* and *gad2* encode for glutamic acid decarboxylases, which are necessary for the synthesis of GABA from glutamate). We found that in both *tal1* and *gata3* mutants, *gad* was expressed by normal numbers of cells in row 1 (KA″ region), but almost no cells in rows 2 and 3 (KA′ region; Figures 2, 7A–F; Supplementary Tables 3–5). There was also a reduction in the number of GABAergic cells in row 4 and above (V2b region), although this reduction was much more pronounced in *tal1* mutants than in *gata3* mutants (Figures 2, 7A–F; Supplementary Table 3). In contrast, there were almost no *gad*-expressing row 1 cells in *gata2a* mutants (KA″ region) and there was also a small, but statistically significant, reduction in the number of *gad*-expressing cells in row 2 and row 4 and above (KA′ and V2b regions) (Figures 2, 7G–I; Supplementary Table 3). In agreement with these single mutant data, almost all *gad*-expressing cells were lost in the spinal cords of *tal1;gata2a* double mutants (Supplementary Figure 3).

KA″ neurons express *urp1* (Quan et al., 2015) and KA′ neurons express *sst1.1* neuropeptides (Djenoune et al., 2017). We found that *sst1.1* expression was lost in both *tal1* and *gata3* mutants (Figures 2, 7J–M) but there were normal numbers of *sst1.1*-expressing KA′ neurons in *gata2a* mutants (data not shown). In contrast, *urp1* expression was lost in *gata2a* mutants (Figures 7N,O) but there were normal numbers of *urp1*-expressing KA″ cells in *gata3* and *tal1* mutants (data not shown).

Taken together, these data suggest that KA″ neurons with appropriate neurotransmitter and neuropeptide phenotypes are not found in *gata2a* mutants, KA′ neurons with appropriate neurotransmitter and neuropeptide phenotypes are not found in either *tal1* or *gata3* mutants and fewer than normal GABAergic V2b neurons are found in all three of these mutants. However, these data also suggest that KA″ neurons develop normally in *tal1* and *gata3* mutants and KA′ neurons develop relatively normally in *gata2a* mutants. There is a small reduction in the number of KA′ cells expressing *sox1a* and *gad* and a very small reduction in the number of these cells expressing *tal1*, but all of the other genes examined are expressed normally in *gata2a* mutants.

### Tal1 and gata3 are required for *pkd2l1* expression in the KA′ region and gata2a is necessary for *pkd2l1* expression in the KA″ region of the spinal cord

**Figure 8:**
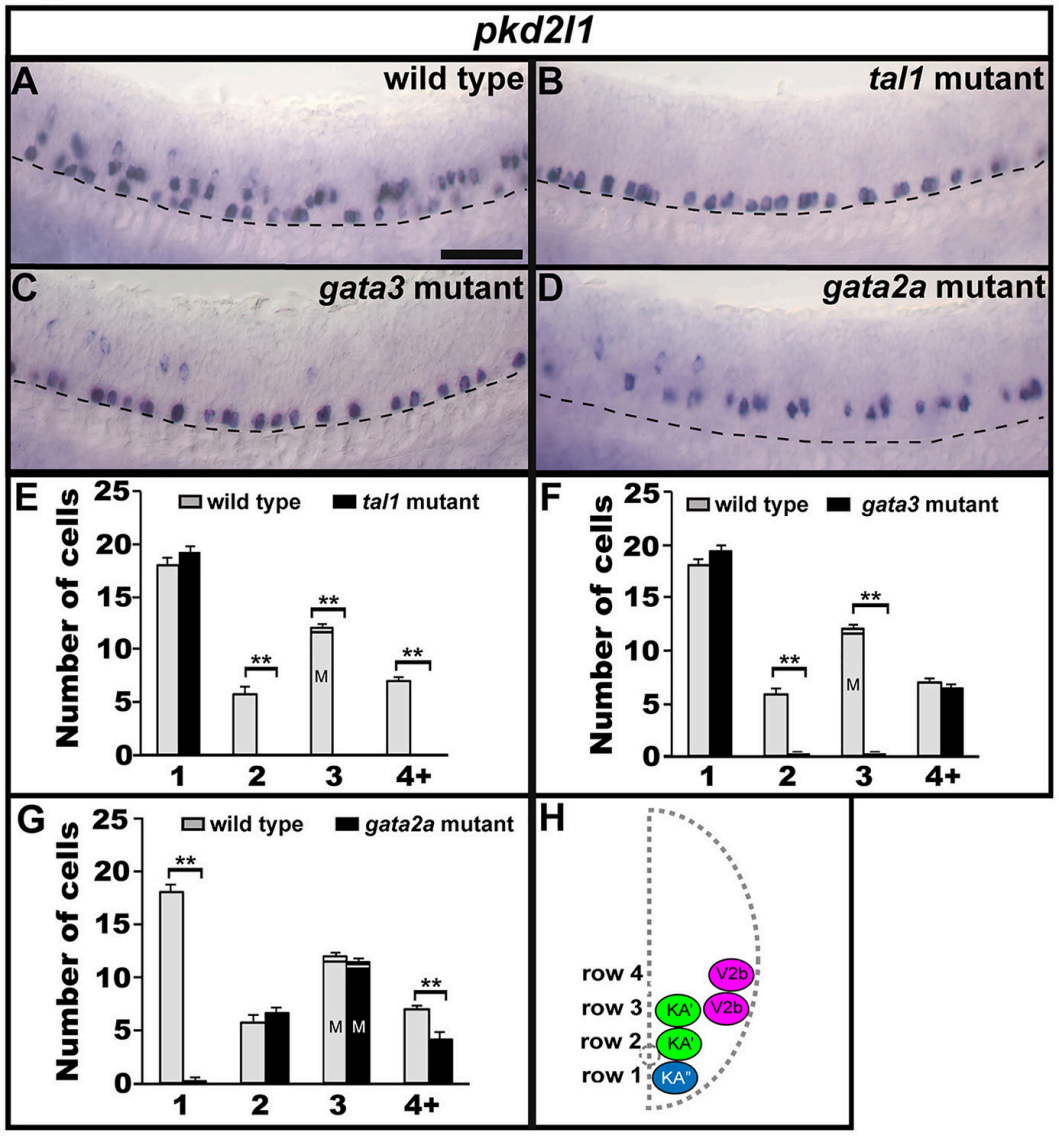
Expression of *pkd2l1* in *tal1, gata3*, and *gata2a* mutants. Lateral views of *pkd2l1* expression in 24 h WT sibling **(A)** and *tal1* **(B)**, *gata3* **(C)**, and *gata2a* **(D)** mutants. Dorsal, top; anterior, left. Scale bar = 50 microns. Dashed lines indicate ventral limit of spinal cord. The approximate proportions of medial and lateral row 3 cells are indicated by horizontal lines separating the number of medially-located cells (bottom and indicated with an “M”) from the number of laterally-located cells (top and indicated with a “L”). Mean number of cells expressing *pkd2l1* in each D/V row of spinal cord region adjacent to somites 6–10 in WT and mutant embryos **(E–G)**. All counts are an average of at least 4 embryos. Error bars indicate SEM. Statistically significant comparisons are indicated with brackets and stars. ^**^P < 0.01. For *P*-values see Supplementary Table 3. Cross-sectional schematic indicating positions of KA″, KA′, and V2b neurons **(H)**.

The non-selective cation-channel gene *pkd2l1* is expressed in both populations of KA neurons in all vertebrates examined so far (Djenoune et al., 2014; Petracca et al., 2016; England et al., 2017) and experiments in zebrafish suggest that Pkd2l1 is crucial for correct KA neuronal function in locomotor circuitry (Bohm et al., 2016). We also find *pkd2l1* expression in occasional V2b neurons (Figure 8A; England et al., 2017). In *tal1* mutants, *pkd2l1* was expressed in normal numbers of cells in row 1 but its expression was lost in the rest of the spinal cord (Figures 2, 8B,E; Supplementary Table 3). Similarly, in *gata3* mutants, *pkd2l1* was expressed in normal numbers of row 1 cells but was not expressed in row 2. However, in contrast to *tal1* mutants, the number of *pkd2l1*-expressing cells in row 4 and above in *gata3* mutants was similar to WT embryos (Figures 2, 8C,F; Supplementary Table 3). Finally, in *gata2a* mutants, there was no change in the number of *pkd2l1*-expressing cells in rows 2 or 3, but expression of *pkd2l1* was almost eliminated in row 1 and there was a slight reduction in the number of *pkd2l1*-expressing cells in row 4 and above (Figures 2, 8D,G; Supplementary Table 3). In agreement with these single mutant data, all *pkd2l1*-expressing cells were lost in the spinal cords of *tal1;gata2a* double mutants (Supplementary Figure 3). As with the neurotransmitter and neuropeptide data discussed above, these results suggest that KA″ neurons with appropriate functional properties are not found in *gata2a* mutants and KA′ neurons with appropriate functional properties are not found in either *tal1* or *gata3* mutants. These data also suggest that KA′ neurons develop normally in *gata2a* mutants and KA″ neurons develop normally in *tal1* and *gata3* mutants.

### KA″ neurons have normal axonal projections in *tal1* and *gata3* mutants and KA′ axonal trajectories are unchanged in *gata2a* mutants

**Figure 9:**
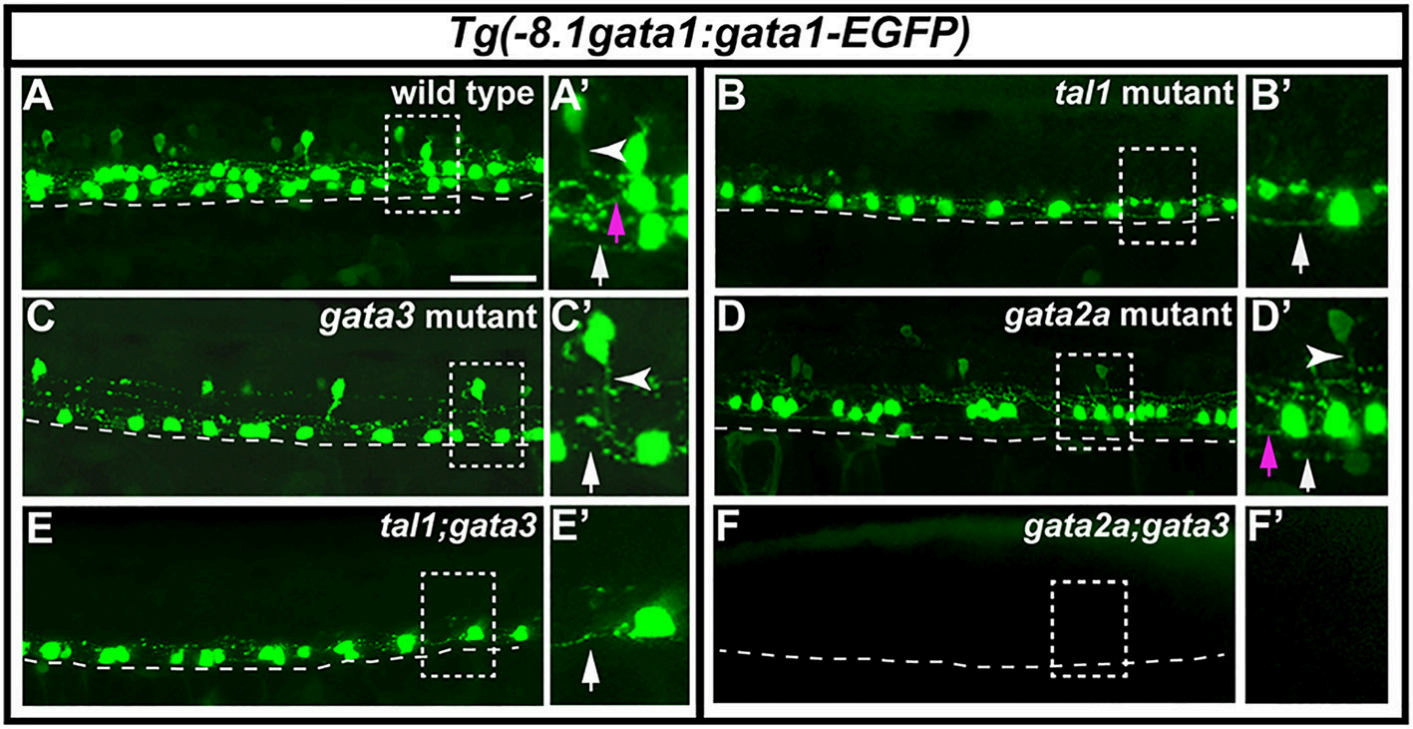
Expression of *Tg(-8.1gata1:gata1-EGFP)* in single and double mutants. Lateral views of 30 h *Tg(-8.1gata1:gata1-EGFP)* WT sibling **(A)** and single and double mutants as indicated **(B–F)**. Dorsal, top; anterior, left. Dashed rectangle regions in main panels **(A–F)** are shown in magnified view in adjacent **(A**′**–F**′**)** panels. Dashed lines indicate ventral limit of spinal cord. White arrows indicate KA″ axons, magenta arrows indicate KA′ axons and white arrowheads mark V2b axons. KA″ and KA′ axons are ipsilateral and ascending whereas V2b axons are ipsilateral descending. KA″ neurons appear to form normally and extend WT-like axonal projections in *tal1;gata3* double mutants **(E,E**′**)**. In contrast, even though GFP-expressing V2b cells are present in both *gata3* **(C,C**′**)** and *gata2a* **(D,D**′**)** single mutants there are no GFP-expressing V2b cells in *gata2a;gata3* double mutants **(F,F**′**)**. Scale bar = 50 microns.

To investigate if *tal1, gata3*, or *gata2a* are required for correct axonal morphology of KA and/or V2b neurons we generated mutant fish transgenic for *Tg(-8.1gata1:gata1-EGFP)*, which labels all KA and some V2b neurons (Batista et al., 2008). In *tal1* mutants, KA″ neurons express GFP and have normal WT-like axonal projections that are ipsilateral and ascend toward the brain (Figure 9B). However, there is no expression of GFP more dorsally in the spinal cord, in either the KA′ or V2b region (Figure 9B). In *gata3* mutants, both V2b and KA″ neurons express GFP and appear to have WT-like axonal projections. The KA″ axons are ipsilateral and ascending and the V2b axons extend ventrally to the midline and then descend ipsilaterally (Figure 9C). However, there are no GFP-labeled KA′ cells (Figure 9C). In *gata2a* mutants, both KA′ and V2b neurons express GFP and have WT-like axonal projections but only a very small number of GFP-positive KA″ neurons remain, although these also have normal axonal projections (Figure 9D; it is also possible that these are KA′ neurons that have moved ventrally in the absence of KA″ neurons). These results, like the data discussed above, suggest that KA′ neurons develop normally in *gata2a* mutants as do KA″ neurons in *tal1* and *gata3* mutants.

### Do *tal1, gata3*, and/or *gata2a* act redundantly in KA and/or V2b development?

Taken together, our results so far suggest that KA″ neurons develop normally in *tal1* and *gata3* mutants as do KA′ neurons in *gata2a* mutants. While we cannot rule out that there are abnormal phenotypes that we have not detected, all of the genes that we have examined are expressed normally in the cells in question, including both early-expressed transcription factor genes and later-expressed genes that encode functional properties of these neurons. In addition, the axons of these neurons follow their normal trajectories. Our analyses also suggest that some V2b neurons develop normally in *gata2a* and *gata3* mutants. However, it was possible that *tal1* and *gata3* might act redundantly in KA″ neurons and/or that *gata2a* and *gata3* might compensate for each other's loss in V2b neurons. Therefore, we examined these cells in the respective double mutants.

**Figure 10:**
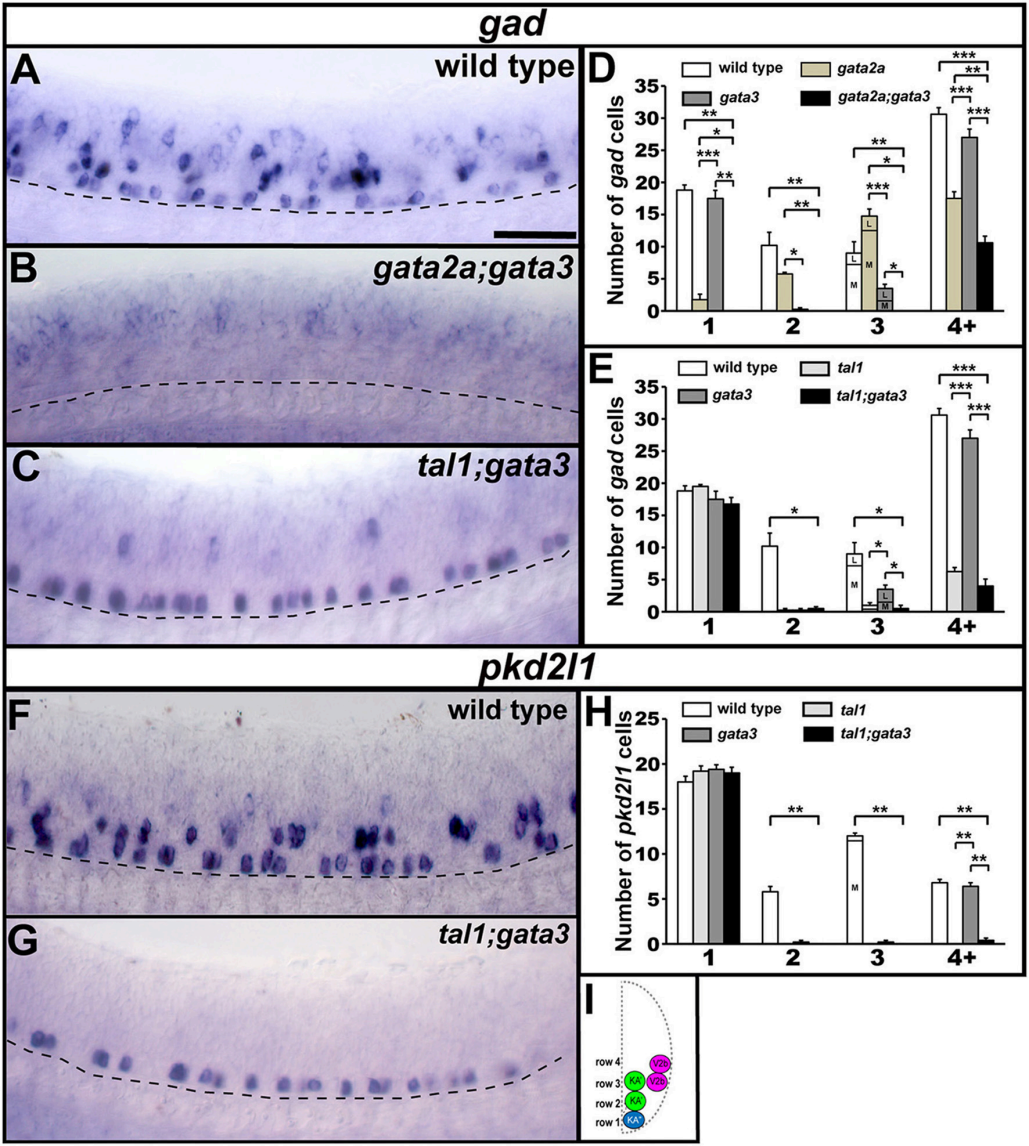
*gad* and *pkd2l1* expression in double mutant embryos. Lateral views of *gad* **(A–C)** or *pkd2l1* **(F,G)** expression in 24 h WT sibling and double mutant embryos as indicated. Dorsal, top; anterior, left. Scale bar = 50 microns. Dashed lines indicate ventral limit of spinal cord. The approximate proportions of medial and lateral row 3 cells are indicated by horizontal lines separating the number of medially-located cells (bottom and indicated where there is space with an “M”) from the number of laterally-located cells (top and indicated where there is space with a “L”). Mean number of cells expressing *gad* or *pkd2l1* in each D/V row of precisely-defined spinal cord region adjacent to somites 6–10 in WT and single and double mutant embryos **(D,E,H)**. All counts are an average of at least 4 embryos. Error bars indicate SEM. Statistically significant comparisons are indicated with brackets and stars. ^***^P < 0.001, ^**^P < 0.01, ^*^P < 0.05. In **(E)**, there is no statistically significant difference between any row 1 values or between *tal1* single mutants and *tal1;gata3* double mutants in row 4+. In **(H)**, there is no statistically significant difference between any row 1 values. For *P*-values see Supplementary Table 3. Cross-sectional schematic indicating positions of KA″, KA′, and V2b neurons **(I)**.

To test if *tal1* and *gata3* act redundantly in KA″ neuronal development we examined functional properties of KA″ neurons in *tal1*;*gata3* double mutants. Using *Tg(-8.1gata1:gata1-EGFP)* we found that, just like in single mutants, KA″ neurons have normal ipsilateral ascending axonal projections in *tal1*;*gata3* double mutants (Figure 9E). In addition, KA″ neurons are still GABAergic and express *pkd2l1* normally in *tal1*;*gata3* double mutants (*gad*-expressing and *pkd2l1*-expressing cells are present in normal numbers in the KA″ region; Figures 10C,E,G,H; Supplementary Table 7). This suggests that *tal1* and *gata3* are not required, either individually or redundantly, for the correct development of KA″ neurons.

To test whether *gata2a* and *gata3* act redundantly in V2b cells we examined *gata2a*;*gata3* double mutants. Using *Tg(-8.1gata1:gata1-EGFP)* we could not detect any GFP-expressing V2b cells (Figure 9F; Supplementary Table 7) in these double mutants. In addition, there were considerably fewer *gad*-expressing cells in the V2b spinal cord region of *gata2a*;*gata3* double mutants than there were in either single mutant (Figures 10B,D). Unlike the *Tg(-8.1gata1:gata1-EGFP)* experiment, some *gad*-expressing cells remain in double mutants, but these are likely be more dorsal GABAergic spinal neurons such as V1 cells or dI4 or dI6 cells (Batista and Lewis, 2008; Hilinski et al., 2016; Juárez-Morales et al., 2016). These results suggest that *gata2a* and *gata3* are required either additively or redundantly in V2b neurons.

### In *gata2a* mutants KA″ cells may transfate into V3 neurons

**Figure 11:**
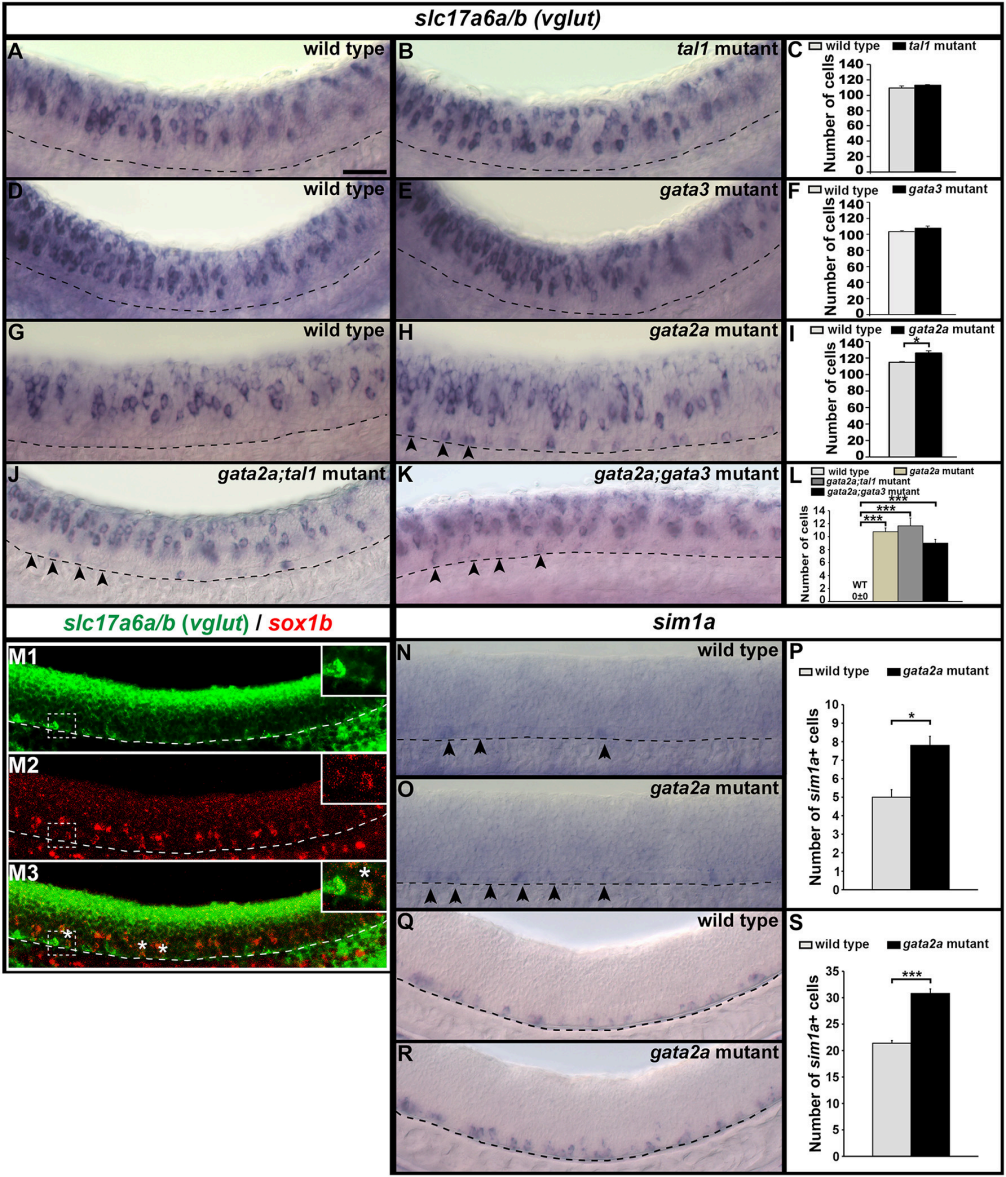
Expression of *slc17a6a/b* and *sim1a* in mutant embryos. Lateral views of *slc17a6a/b (vglut)* **(A,B,D,E,G,H,J,K)**, *slc17a6a/b* (green) and *sox1b* (red) **(M)**, and *sim1a* **(N,O,Q,R)** expression in WT sibling and mutant embryos as indicated. Dorsal, top; anterior, left. All embryos are 24 h except **(M,Q,R)**, which are 32 h. Arrowheads indicate glutamatergic cells **(H,J,K)** or *sim1a*-expressing cells **(N,O)** in the KA″ region. Scale bar = 50 microns. Dashed lines indicate ventral limit of spinal cord. White boxes in **(M)** are single confocal magnified views of dotted white box. White stars indicate double-labeled cells. Mean number of cells expressing these genes in spinal cord region adjacent to somites 6–10 in WT and mutant embryos **(C,F,I,L,P,S)**. Panels **(C,F,I,P,S)** show counts for all dorsal-ventral rows. Panel **(L)** shows counts for just row 1 and hence, the value for WT embryos is zero. All counts are an average of at least 4 embryos. Error bars indicate SEM. Statistically significant comparisons are indicated with brackets and stars. ^***^P < 0.001, ^*^P < 0.05. For *P*-values see Supplementary Table 6.

Our data suggest that KA″ cells do not die in *gata2a* mutants, as there is no increase in the expression of a cell death marker in these mutants (Figure 6F) and there is no change in the number of row 1 cells expressing *sox1b* or *tal2* (Figures 2, 3Q–S, 5Q–S). However, KA″ cells do not develop correctly in these mutants as they lose expression of all of the other KA″ genes that we have examined and they do not develop correct KA″ functional characteristics. As these cells are no longer GABAergic, we tested whether they become glutamatergic (excitatory) and we found a statistically significant increase in the number of cells expressing the glutamatergic markers *slc17a6a/b* (*slc17a6a* and *slc17a6b* encode glutamate transporters, see section Materials and Methods; Figures 11G–I; Supplementary Table 6). In contrast, *tal1* and *gata3* mutants had no increase in the number of glutamatergic cells (Figures 11A–F). While WT embryos at 24h had no glutamatergic cells in row 1, *gata2a* mutants had approximately 11 glutamatergic cells in this row in the spinal cord region adjacent to somites 6–10 (Figures 11G,H,L; Supplementary Table 6).

KA″ cells arise from the same progenitor domain (p3) as glutamatergic V3 interneurons (Park et al., 2004). V3 neurons, like KA″ cells, express *tal2* (Schäfer et al., 2007; Yang et al., 2010). However they also express a unique V3 marker *sim1a* (Schäfer et al., 2007; Yang et al., 2010). In zebrafish, most V3 neurons form later than KA neurons. Earlier reports suggested that *sim1a* is not expressed until 36h (Schäfer et al., 2007; Yang et al., 2010). However, we detected a few scattered *sim1a*-expressing cells in row 1 in 24 h WT embryos (Figures 11N,P) and the number of these cells was statistically significantly increased in *gata2a* mutants at both 24 h (Figures 11O,P) and at 32 h (Figures 11Q–S), suggesting that at least some KA″ cells are transfating into V3 neurons or acquiring a hybrid V3/KA″ identity.

To establish whether V3 neurons also express *sox1b* we performed double-labeling experiments for *sox1b* and *slc17a6a/b* at 32 hpf when V3 cells are present in larger numbers. We found several cells in row 1 that co-express these two genes, suggesting that V3 cells do indeed express *sox1b* (Figure 11M).

We had already established that KA″ neurons form normally in *tal1* and *gata3* single and double mutants. However, it was still theoretically possible that Tal1 and/or Gata3 might act partially redundantly with Gata2a to repress the V3 fate in KA″ cells. To test this, we examined the number of V3 cells (glutamatergic cells in row 1) in both *gata2a;tal1* and *gata2a;gata3* double mutants. However, there was no statistically significant change in the number of V3 cells in either double mutant, compared to *gata2a* single mutants (Figures 11J–L).

### KA′ cells may fail to differentiate in *gata3* and *tal1* mutants

**Figure 12:**
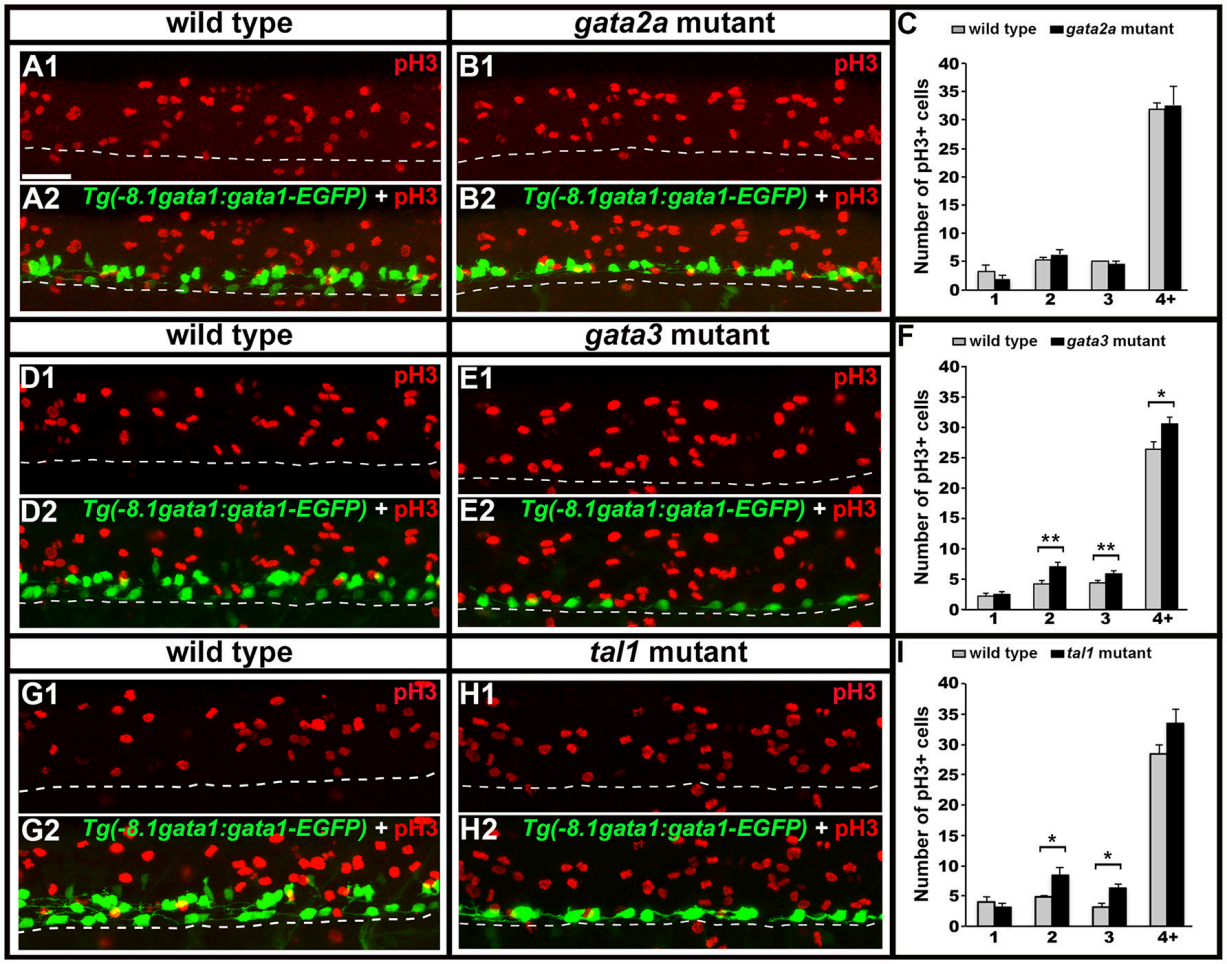
Phospho-histone H3 (pH3) immunohistochemistry in *tal1, gata3*, and *gata2a* mutants. Lateral views of pH3 (red) and GFP (green) immunohistochemistry in 24 h *Tg(-8.1gata1:gata1-EGFP)* WT sibling and mutant embryos as indicated **(A,B,D,E,G,H)**. Top panel (1) in each case shows just the red channel and bottom panel (2) shows the merged image of both green and red channels. *Tg(-8.1gata1:gata1-EGFP)* embryos were used to help orientate to particular rows/cell type regions in the ventral spinal cord. Dorsal, top; anterior, left. Scale bar = 50 microns. Dashed lines indicate ventral limit of spinal cord. Mean number of pH3-positive cells in each D/V row of spinal cord region adjacent to somites 6–10 in WT and mutant embryos **(C,F,I)**. All counts are an average of at least 3 embryos. Cell rows were assigned based on average cell diameters. Error bars indicate SEM. Statistically significant comparisons are indicated with brackets and stars. ^*^P < 0.05, ^**^P < 0.01. For *P*-values see Supplementary Table 6.

**Figure 13:**
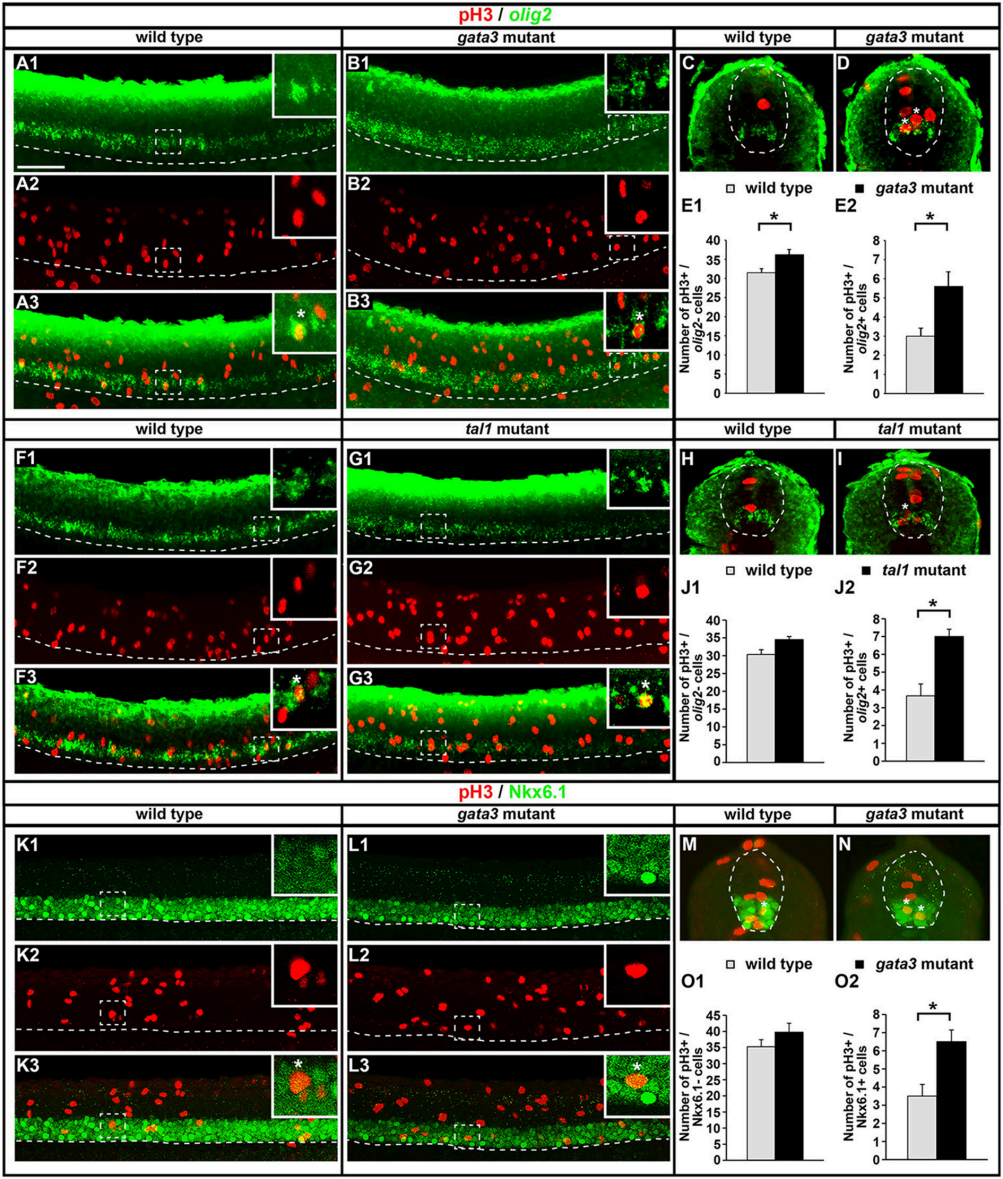
Expression of pH3 and either *olig2* or Nkx6.1 in *tal1* and *gata3* mutants. Lateral **(A,B,F,G,K,L)** and cross-sectional **(C,D,H,I,M,N)** views of pH3 (red) and either *olig2* or Nkx6.1 (green) expression in WT embryos **(A,C,F,H,K,M)**, *gata3* mutants **(B,D,L,N)**, or *tal1* mutants **(G,I)** at 24 h. Dorsal, top; in lateral views, anterior, left. White boxes in top right hand corners of lateral views are single confocal plane magnified views of the area indicated with a white dotted box. White stars indicate double-labeled cells. White dashed lines indicate the ventral limit of the spinal cord (lateral views) or the boundary of the spinal cord (cross-sections). For lateral views, single channel and merged images are shown. Scale bar = 50 microns. Mean number of pH3-positive; *olig2*-negative cells **(E1,J1)** and pH3-positive; *olig2*-positive cells **(E2,J2)** adjacent to somites 6–10 in WT and mutant embryos. Mean number of pH3-positive; Nkx6.1-negative cells **(O1)** and pH3-positive; Nkx6.1-positive cells **(O2)** adjacent to somites 6–10 in WT and mutant embryos. All counts are an average of at least 3 embryos. Cell rows were assigned based on average cell diameters. Error bars indicate SEM. Statistically significant comparisons are indicated with brackets and stars. ^*^P < 0.05. For *P*-values see Supplementary Table 6.

KA′ cells do not appear to be dying or transfating into neighboring cell types in *gata3* or *tal1* mutants, but they also do not express any of the normal KA′ markers that we have examined. Therefore, we tested if these cells might be failing to differentiate, by quantifying mitotically-active cells with phospho-histone H3 (pH3) staining. As expected, we found no statistically significant difference in the number of pH3-positive nuclei in *gata2a* mutants compared to WT embryos (Figures 12A–C). However, there was an increase in the number of pH3-positive nuclei in both *tal1* and *gata3* mutants. The number of pH3-positive cells in row 1 was unchanged, consistent with normal differentiation of KA″ neurons in these mutants but the number of pH3-positive cells was increased in rows 2 and 3 (the KA′ and pMN progenitor domain; Figures 12D–I), suggesting that KA′ cells may be failing to exit the cell cycle. Double labeling experiments with pH3 and *olig2* (Park et al., 2002) further confirmed these results. Both *gata3* and *tal1* mutants have a statistically significant increase in the number of pH3-positive, *olig2*-positive cells compared to WT embryos (Figures 13A–J; Supplementary Table 6), demonstrating that there is an increase in the number of mitotically-active cells in the pMN progenitor domain, from which KA′ cells are usually generated. *gata3* mutants also have a small, but statistically significant, increase in the number of pH3-positive, *olig2*-negative cells compared to WT embryos (Figures 13A–E; Supplementary Table 6), which is consistent with the small, but statistically significant, increase in the number of pH3-positive cells in row 4 and above in these mutants (Figure 12F; Supplementary Table 6). Our double labeling experiments with Nkx6.1 suggest that these “extra” mitotically-active cells that are not within the pMN progenitor domain, are instead within the p2 progenitor domain, as *gata3* mutants had a statistically significant increase in the number of pH3-positive, Nkx6.1-positive cells compared to WT embryos but there was no difference in the number of pH3-positive, Nkx6.1-negative cells between mutants and WTs (Figures 13K–O; Supplementary Table 6).

## Discussion

To understand how correct neural circuits form during development we need to determine how the distinct neurons that constitute these circuits are specified. In this paper, we used mutant zebrafish to test the functions of three transcription factors, Tal1, Gata2a, and Gata3, in multiple aspects of KA″, KA′, and V2b spinal neuron specification and development. These include correct expression of transcription factor genes; cell viability; differentiation from progenitor cells and development of essential functional characteristics, such as GABAergic neurotransmitter phenotypes, appropriate axonal trajectories, expression of appropriate neuropeptide genes and expression of a crucial channel gene *pkd2l1*. Our data suggest that, in zebrafish, Gata2a is required to specify a KA″ and repress a V3 fate in cells that would normally develop into KA″ neurons and Gata3 and Tal1 are required for KA′ neurons to differentiate from progenitor cells. In addition, all three of these transcription factors are required for later stages of V2b neuron differentiation.

### Gata2a is required for specification of KA″ neurons

In *gata2a* mutants, there is no longer any expression of KA″ markers *gata3, tal1, sox1a, gad, pkd2l1* or *urp1* in the most ventral row of the spinal cord (row 1), which is where KA″ cells normally form (Figures 2, 3K–P, 5N–P, 7G–I,N,O 8D,G). In addition, only very occasional cells in this row express *Tg(-8.1gata1:gata1-EGFP)* (Figure 9D) and it is possible that these may be KA′ neurons that have moved ventrally in the absence of KA″ neurons. However, normal numbers of cells still express both *sox1b* and *tal2* in the KA″ region of these mutants (Figures 3Q–S, 5Q–S), suggesting that cells that would normally have developed into KA″ cells are still differentiating in normal numbers and are still expressing both of these genes. Consistent with this, we didn't observe any increase in cell death in this region of the spinal cord or any increase in the number of mitotically-active cells (Figures 6F, 12A–C). *tal2* is also expressed by glutamatergic V3 cells, which usually form later than KA″ cells in row 1 (Schäfer et al., 2007; Yang et al., 2010). Our data suggest that V3 cells also express *sox1b* (Figure 11M). This suggests that the row 1 *tal2*- and *sox1b*-expressing cells in *gata2a* mutants might be ectopic V3 cells. Consistent with this, we found an increase in the number of glutamatergic cells in row 1 and the number of cells expressing the V3 specific gene *sim1a* in these mutants (Figures 11L,P,S). Taken together, these data suggest that Gata2a is required for the specification of KA″ neurons, and that in *gata2a* mutants, cells that would have become KA″ neurons acquire at least some V3 characteristics. Gata2a may actively repress the V3 fate in these cells, or, alternatively, it is possible that the V3 fate is a default fate for row 1 neurons. Gata2 is also expressed by amniote CSF-cN/KA neurons (Petracca et al., 2016), but its function in these cells has not yet been examined. Unlike in zebrafish, where KA″ neurons are some of the earliest neurons to form in the spinal cord, in mouse CSF-cN/KA neurons form after most other spinal cord neurons (Petracca et al., 2016). Therefore, in future work, it would be interesting to test whether Gata2 function in ventral CSF-cN/KA″ neurons is conserved between mouse and zebrafish.

### Gata3 and tal1 are required for differentiation of KA′ neurons

In *gata3* and *tal1* mutants, there are almost no row 2 or medial row 3 cells that express KA′ genes *gata2a, gata3, tal1, tal2, sox1a, sox1b, GAD, pkd2l1*, or *sst1.1* and there are no *Tg(-8.1gata1:gata1-EGFP*)-positive KA′ neurons, suggesting that KA′ neurons do not form in these mutants (Figures 1–3, 5–9). Occasional row 2 cells that express one of these genes may be slightly more dorsally-located KA″ neurons. In theory, it is possible that cells expressing KA′ markers are not found in the normal KA′ region because the location of KA′ neurons has changed in these mutants. However, our data does not support this hypothesis, as there is no corresponding increase in the number of cells expressing any of these genes in any other spinal cord region. In addition, we do not think that KA′ cells are dying or transfating into motoneurons or KA″, V2a, or V2b neurons in these mutants, as there is no increase in the number of cells expressing cell death markers or markers of these neighboring cell types (Figures 1–3, 5–9). Instead, our data suggest that the most likely explanation for the loss of KA′ neurons is that these cells are not differentiating, as there is an increase in the number of mitotically-active precursor cells in the pMN progenitor domain, from which KA′ cells are normally generated, in both *gata3* and *tal1* mutants (Figures 12, 13). Gata3 is also expressed by mouse CSF-cN/KA neurons (Petracca et al., 2016), but it is not yet known if this is also the case for Tal1 and the function of Gata3 in these cells has not yet been examined in mouse. Therefore, in future work, it would be interesting to test if the functions of these transcription factors in dorsal CSF-cN/KA′ neurons are conserved between mouse and zebrafish.

### Gata2a, gata3, and tal1 are all required for normal development of at least some V2b neurons

*gata2a, gata3* and *tal1* mutants all have distinct phenotypes in spinal cord row 4 and above, which corresponds to the region of the spinal cord where V2b cells are located. In each mutant, several V2b genes are expressed by a reduced number of cells in this region, although in each case at least one V2b gene is expressed in a normal number of cells (Figures 1–3, 5, 7–9). Our data suggest that the V2b cells with mutant phenotypes are not dying, or acquiring a hybrid or new identity as there is no increase in the number of cells expressing a cell death marker, or markers of motoneurons or KA, V2a, V1, or V0v neurons (we see no increase in the number of cells expressing KA/V2b genes, Islet1/2, *vsx1, gad*, or *slc17a6a/b*, other than the increased number of *slc17a6a/b*-expressing cells in row 1 discussed above; Figures 1–3, 5–8, 11). Our data also suggest that V2b cells are not mis-located in any of these mutants (for example present in more ventral KA spinal cord regions) as there is no increase in the number of cells expressing V2b genes in any other spinal cord region. Finally, there is no change in the number of mitotically-active cells in the V2b region in *tal1* or *gata2a* mutants (Figure 12). We did detect a small, but statistically significant, increase in the number of mitotically-active cells in the V2b region of the spinal cord in *gata3* mutants (Figure 12F). However, if *gata3* is required for some V2b cells to differentiate from late progenitor cells, similar to its function in KA′ cells, it is surprising that normal numbers of cells expressing *tal2* and *sox1a* are present in the V2b region (Figures 2, 3J, 5J). In addition, given that V2a and V2b cells are usually generated as sister cells from the same progenitor cell (Del Barrio et al., 2007; Peng et al., 2007; Batista et al., 2008; Kimura et al., 2008; Joshi et al., 2009), if some V2b neurons are failing to differentiate, it is surprising that we do not also see a reduction in the number of V2a cells in these mutants (Figures 6P–X). Given that we also observed a slight increase, that was not statistically significant, in the number of mitotically-active cells in the V2b region of the spinal cord in *tal1* mutants (Figure 12I), an alternative explanation might be that there is an increase in the number of mitotically-active cells in the V2b region, because a small number of the “extra” KA′ progenitor cells have been displaced dorsally in these mutants.

*tal1* mutants have the most severe abnormal V2b phenotype, with a complete loss of *Tg(-8.1gata1:gata1-EGFP)*-positive V2b neurons and a severe reduction in the number of GABAergic cells in row 4 and above, suggesting that at least most V2b neurons require Tal1 to develop correct functional characteristics (Figures 7C, 9B, 10E). It is possible that there are no GABAergic V2b cells in *tal1* mutants and that the small number of remaining GABAergic cells in row 4 and above are V1 neurons, a subset of which are GABAergic or more dorsal GABAergic cells (Batista and Lewis, 2008). In addition, in the V2b region of *tal1* mutants there are no cells expressing *tal2*, almost no cells expressing *sox1b* and a reduced number of cells expressing *sox1a* (Figures 1N, 2, 5D,G). In contrast, there are normal numbers of cells expressing *gata2a* and *gata3* in this spinal cord region, which is consistent with V2b cells at least starting to differentiate and not dying.

The *gata2a* and *gata3* V2b mutant phenotypes are less severe. However, *gata2a;gata3* double mutants have a much more severe V2b phenotype than either *gata2a* or *gata3* single mutants, suggesting that these genes either have partially-redundant functions in this cell type or they are each required in a different subset of V2b cells. Like *tal1* single mutants, *gata2a;gata3* double mutants lose expression of *Tg(-8.1gata1:gata1-EGFP)* in the V2b region (Figure 9F) and there is a more severe reduction in the number of row 4 and above GABAergic cells in these double mutants than in either single mutant (Figure 10D). As with *tal1* mutants, it is likely that at least most of the remaining dorsal GABAergic cells in *gata2a;gata3* double mutants are V1 or more dorsal cells, suggesting that there may be no GABAergic V2b cells in these double mutants.

Taken together, our data strongly suggest that *gata2a, gata3* and *tal1* are not required for V2b neuron differentiation or survival, but that they are instead required for later aspects of V2b neuronal development including the acquisition of correct functional characteristics.

### Differences between V2b cell development in zebrafish and mouse

Interestingly our analyses have uncovered some differences in zebrafish V2b development compared to mouse. In zebrafish, *sox1a* and *sox1b* are expressed by at least most, and probably all, V2b neurons (Figure 4), whereas in mouse, *Sox1* is only expressed by a small subset of V2b cells that turn off expression of Gata3 and develop into V2c cells (Panayi et al., 2010). This suggests that V2c cells may have evolved in tetrapods, perhaps as part of the evolution of different types of locomotion associated with limb-based movement on land (see also similar argument for subsets of V1 cells; Higashijima et al., 2004c; Lewis, 2006).

In addition, while mouse *Gata2* mutants lose all V2 cells (Nardelli et al., 1999; Zhou et al., 2000; Francius et al., 2015), in zebrafish *gata2a* mutants, we found no change in the number of cells expressing the V2a gene *vsx1* (Figures 6V–X), or in the number of V2b cells expressing *tal1, tal2, sox1a* or *sox1b* (Figures 3N–S, 5N–S). There was just a slight reduction in the number of V2b cells expressing *gata3, pkd2l1* or *gad* (Figures 3K–M, 7G–I, 8D,G). This suggests that Gata2 has a different function in V2 neurons in zebrafish compared to mouse, acting later in development and only in V2b cells.

Finally, while expression of several genes, and all of the functional characteristics that we analyzed, were lost in V2b cells in zebrafish *tal1* mutants, unlike in mouse, there was no change in the number of V2b cells expressing *gata2a* and there was no increase in the number of *vsx1*-expressing V2a cells (Figures 1I–K, 6P–R). This suggests that Tal1 function in V2b neurons also differs between zebrafish and mouse and like with Gata2a, in zebrafish, Tal1 is probably required at a later stage of V2b development, after V2a and V2b neurons have been specified from their common progenitor cell (V2a and V2b neurons are sister cells, generated by the final division of p2 progenitor cells; Del Barrio et al., 2007; Peng et al., 2007; Batista et al., 2008; Kimura et al., 2008; Joshi et al., 2009). These differences between mouse and zebrafish are consistent with the idea that while some transcription factor functions are highly conserved between zebrafish and mammalian spinal cord (e.g., Cheesman et al., 2004; Lewis et al., 2005; Batista and Lewis, 2008), there is also some evolutionary plasticity in the functions of other transcription factors in spinal neural development. For example, in zebrafish, unlike in mouse, Evx1 is not required to repress the V1 fate in V0v neurons, possibly because Dbx1 expression persists in zebrafish V0v cells for a short while after they become post-mitotic but is lost in mouse V0v cells (Juárez-Morales et al., 2016). In other examples, loss of Islet1 causes motoneurons to die in mouse but these cells instead change into interneurons in zebrafish (Hutchinson and Eisen, 2006), Nkx6 proteins have slightly different roles in motoneurons in zebrafish and mouse (Hutchinson et al., 2007) and Gli3 has different functions in ventral spinal cord patterning in zebrafish and mouse, probably as a result of the fast development of zebrafish spinal neurons (England et al., 2011). In future studies, it would be interesting to further investigate the evolutionary differences in V2b neuron development identified in this paper and determine where in the vertebrate lineage each of them arose.

### Tal1 and gata3 are probably not necessary for development of KA″ neurons and gata2a is probably not required for KA′ neuron development

Surprisingly, even though *tal1, gata2a*, and *gata3a* are all expressed by KA″, KA′, and V2b neurons, our experiments have not revealed any requirement for *tal1* or *gata3*, either singly or redundantly, in KA″ development or for *gata2a* in KA′ development. The only exception is a very slight reduction in the number of GABAergic and *sox1a*-expressing cells in row 2 in *gata2a* mutants, which could potentially be due to a few KA″ neurons being counted in row 2 in WT embryos in these experiments. In addition, while at least some V2b neurons do not develop normally in each of these three mutants, V2b phenotypes are different in each mutant (Figures 1–3, 5, 7–9), suggesting that these genes may also have distinct functions in this cell type. While we cannot rule out the possibility that cells that appear to develop normally in our experiments have abnormal phenotypes in aspects of KA and V2b neuronal development that we have not assayed, such as synapse formation or dendritic morphology, these results demonstrate that *gata2a, gata3*, and *tal1* each have unique functions in spinal cord development. It is particularly surprising that *gata2a* and *gata3* have distinct functions in KA″ and KA′ neurons as these genes encode highly-related transcription factors and act redundantly in many tissues (Haugas et al., 2016; Home et al., 2017). It is possible that even though each of these three genes is expressed in V2b, KA′, and KA″ cells, each gene is only translated in a subset of these cell types. This could be tested in future studies by developing specific antibodies that work in zebrafish spinal cord, for each of these proteins. A more likely scenario might be that different co-factor proteins interact differentially with these transcription factors in each cell type.

### Most of our mutant data is consistent with earlier morpholino studies

While the functions of Gata2a and Gata3 in KA neuron development had been analyzed previously using morpholino knock-down experiments (Yang et al., 2010), it was important to test these findings using mutants as morpholinos can have non-specific, off-target effects (Kok et al., 2015). Our *gata2a* and *gata3* mutant KA cell phenotypes are mainly consistent with these earlier morpholino knock-down experiments (Yang et al., 2010). However, unlike in *gata2a* morphants, *tal2* expression is not lost in KA″ cells in *gata2a* mutants, although it may be slightly reduced (Figures 3Q–S). There are at least three possible explanations for this discrepancy. First, the *gata2a* morpholino may have caused a non-specific effect in KA″ cells, resulting in levels of *tal2* expression too low to detect. Second, if *tal2* expression is indeed weaker in these cells in the absence of Gata2a, it is possible that these earlier experiments did not detect its expression. Third, it is theoretically possible that the *gata2a* mutant may not be a null allele, and hence may not remove as much Gata2a activity as the morpholino. However, this last hypothesis is extremely unlikely, as even if a truncated Gata2a protein is made in these mutants, the protein would lack both of its zinc fingers (Zhu et al., 2011). In addition, all of the other aspects of the phenotype are similar between the two experiments. Yang and colleagues also reported that *gad* expression in the V2b region was not affected in *gata3* knock-down embryos (Yang et al., 2010), whereas in *gata3* mutants we discovered a slight reduction in the number of V2b cells expressing *gad*. However, this reduction could have easily been missed in the earlier study, as they did not count the number of cells expressing *gad*.

### Tal2 may also be required for correct KA″ cell development

Interestingly, Yang and colleagues also analyzed morpholino knock-down of *tal2* (Yang et al., 2010). These experiments suggested that Tal2 is required for KA″, but not KA′ cells to become GABAergic, although expression of *gata2a* and *gata3* was unaffected in the KA″ cells. While this is a more subtle phenotype than any of our mutant phenotypes, it is striking that these data suggest that *tal2* is required specifically in KA″ but not KA′ cells, which is the opposite of *tal1*, in the same way that *gata2a* is required specifically in KA″ but not KA′ cells, which is the opposite of *gata3*. Along with the similarity of the *tal1* and *gata3* mutant phenotypes in KA′ cells, this suggests that distinct pairs of Gata and Tal proteins may be required either as part of a complex, or in parallel, for correct development of KA″ and KA′ cells. This could be tested in future work by creating *tal2* single and double mutants. There is a precedence for the idea that Tal and Gata proteins may function in a protein complex, as in mouse V2 cells, Tal1, and Gata2 form a protein complex with LMO4 and NLI (Joshi et al., 2009).

## Conclusions

Taken together, the data in this paper provide substantial new insights into the spinal cord functions of *tal1, gata2a*, and *gata3* and significantly enhance our understanding of how KA (CSF-cN) and V2b specification and differentiation are genetically regulated in zebrafish. Our analyses confirm many aspects of the KA phenotypes of previous morpholino knock-downs of Gata2a and Gata3. We also identify, for the first time in any vertebrate, a crucial function for Tal1 in KA′ neuron differentiation and Gata3 in V2b neuron development. Finally, we show that while Gata2a and Tal1 are also required for correct V2b neuron development in zebrafish, these transcription factors have different, later functions in these cells in zebrafish than they do in mouse.

## Author contributions

LA and SB: performed most of the experiments for this paper and prepared initial versions of most of the figures and tables; KK: performed initial analyses of *tal1* and *gata3* mutants; SE: performed additional experiments requested by reviewers and helped to prepare figures and tables for the paper and performed statistical analyses; CV: performed some of the genotyping of mutants and expression analyses; KL: directed the study and wrote the paper with help from the other authors. All authors read and commented on drafts of the paper and approved the final version.

### Conflict of interest statement

The authors declare that the research was conducted in the absence of any commercial or financial relationships that could be construed as a potential conflict of interest.

## References

[B1] Abràmoff M. D. Magalhães P. J. Ram S. J. (2004). Image processing with imageJ. Biophoton. Int. 11, 36–41.

[B2] Agduhr E. (1922). Über ein Zentrales Sinnesorgan bei den Vertebraten. Z. Anat. Entwicklungs 66, 223–360. 10.1007/BF02593586

[B3] Al-Mosawie A. Wilson J. M. Brownstone R. M. (2007). Heterogeneity of V2-derived interneurons in the adult mouse spinal cord. Eur. J. Neurosci. 26, 3003–3015. 10.1111/j.1460-9568.2007.05907.x 18028108

[B4] Armant O. Marz M. Schmidt R. Ferg M. Diotel N. Ertzer R.. (2013). Genome-wide, whole mount *in situ* analysis of transcriptional regulators in zebrafish embryos. Dev. Biol. 380, 351–362. 10.1016/j.ydbio.2013.05.006 23684812 PMC4351915

[B5] Barber R. P. Vaughn J. E. Roberts E. (1982). The cytoarchitecture of GABAergic neurons in rat spinal cord. Brain Res. 238, 305–328. 10.1016/0006-8993(82)90107-X 7046873

[B6] Batista M. F. Lewis K. E. (2008). Pax2/8 act redundantly to specify glycinergic and GABAergic fates of multiple spinal interneurons. Dev. Biol. 323, 88–97. 10.1016/j.ydbio.2008.08.009 18761336 PMC2849013

[B7] Batista M. F. Jacobstein J. Lewis K. E. (2008). Zebrafish V2 cells develop into excitatory CiD and Notch signalling dependent inhibitory VeLD interneurons. Dev. Biol. 322, 263–275. 10.1016/j.ydbio.2008.07.015 18680739

[B8] Bernhardt R. R. Patel C. K. Wilson S. W. Kuwada J. Y. (1992). Axonal trajectories and distribution of GABAergic spinal neurons in wildtype and mutant zebrafish lacking floor plate cells. J. Comp. Neurol. 326, 263–272. 10.1002/cne.903260208 1479075

[B9] Bohm U. L. Prendergast A. Djenoune L. Nunes Figueiredo S. Gomez J. Stokes C.. (2016). CSF-contacting neurons regulate locomotion by relaying mechanical stimuli to spinal circuits. Nat. Commun. 7, 10866. 10.1038/ncomms10866 26946992 PMC4786674

[B10] Britz O. Zhang J. Grossmann K. S. Dyck J. Kim J. C. Dymecki S. (2015). A genetically defined asymmetry underlies the inhibitory control of flexor-extensor locomotor movements. Elife 4:e04718 10.7554/eLife.04718 PMC4604447 26465208

[B11] Bussmann J. Bakkers J. Schulte-Merker S. (2007). Early endocardial morphogenesis requires Scl/Tal1. PLoS Genet. 3:e140. 10.1371/journal.pgen.0030140 17722983 PMC1950955

[B12] Butko E. Distel M. Pouget C. Weijts B. Kobayashi I. Ng K.. (2015). Gata2b is a restricted early regulator of hemogenic endothelium in the zebrafish embryo. Development 142, 1050–1061. 10.1242/dev.119180 25758220 PMC4360177

[B13] Cheesman S. E. Layden M. J. Von Ohlen T. Doe C. Q. Eisen J. S. (2004). Zebrafish and fly Nkx6 proteins have similar CNS expression patterns and regulate motoneuron formation. Development 131, 5221–5232. 10.1242/dev.01397 15456722

[B14] Cheng L. Samad O. A. Xu Y. Mizuguchi R. Luo P. Shirasawa S.. (2005). Lbx1 and Tlx3 are opposing switches in determining GABAergic versus glutamatergic transmitter phenotypes. Nat. Neurosci. 8, 1510–1515. 10.1038/nn1569 16234809

[B15] Concordet J. P. Lewis K. E. Moore J. W. Goodrich L. V. Johnson R. L. Scott M. P.. (1996). Spatial regulation of a zebrafish patched homologue reflects the roles of sonic hedgehog and protein kinase A in neural tube and somite patterning. Development 122, 2835–2846. 8787757 10.1242/dev.122.9.2835

[B16] Craven S. E. Lim K. C. Ye W. Engel J. D. de Sauvage F. Rosenthal A. (2004). Gata2 specifies serotonergic neurons downstream of sonic hedgehog. Development 131, 1165–1173. 10.1242/dev.01024 14973276

[B17] Dale N. Roberts A. Ottersen O. P. Storm-Mathisen J. (1987). The morphology and distribution of 'Kolmer-Agduhr cells', a class of cerebrospinal-fluid-contacting neurons revealed in the frog embryo spinal cord by GABA immunocytochemistry. Proc. R. Soc. Lond,. B Biol. Sci. 232, 193–203. 2892204 10.1098/rspb.1987.0068

[B18] Del Barrio M. G. Taveira-Marques R. Muroyama Y. Yuk D. I. Li S. Wines-Samuelson M.. (2007). A regulatory network involving Foxn4, Mash1 and delta-like 4/Notch1 generates V2a and V2b spinal interneurons from a common progenitor pool. Development 134, 3427–3436. 10.1242/dev.005868 17728344 PMC6329449

[B19] Djenoune L. Desban L. Gomez J. Sternberg J. R. Prendergast A. Langui D.. (2017). The dual developmental origin of spinal cerebrospinal fluid-contacting neurons gives rise to distinct functional subtypes. Sci. Rep. 7:719. 10.1038/s41598-017-00350-1 28389647 PMC5428266

[B20] Djenoune L. Khabou H. Joubert F. Quan F. B. Nunes Figueiredo S. Bodineau L.. (2014). Investigation of spinal cerebrospinal fluid-contacting neurons expressing PKD2L1: evidence for a conserved system from fish to primates. Front. Neuroanat. 8:26. 10.3389/fnana.2014.00026 24834029 PMC4018565

[B21] England S. J. Campbell P. C. Banerjee S. Swanson A. J. Lewis K. E. (2017). Identification and expression analysis of the complete family of zebrafish pkd genes. Front Cell Dev Biol 5:5. 10.3389/fcell.2017.00005 28271061 PMC5318412

[B22] England S. Batista M. F. Mich J. K. Chen J. K. Lewis K. E. (2011). Roles of Hedgehog pathway components and retinoic acid signalling in specifying zebrafish ventral spinal cord neurons. Development 138, 5121–5134. 10.1242/dev.066159 22069186 PMC3210494

[B23] Francius C. Ravassard P. Hidalgo-Figueroa M. Mallet J. Clotman F. Nardelli J. (2015). Genetic dissection of Gata2 selective functions during specification of V2 interneurons in the developing spinal cord. Dev. Neurobiol. 75, 721–737. 10.1002/dneu.22244 25369423

[B24] Gross M. K. Dottori M. Goulding M. (2002). Lbx1 specifies somatosensory association interneurons in the dorsal spinal cord. Neuron 34, 535–549. 10.1016/S0896-6273(02)00690-6 12062038

[B25] Haugas M. Tikker L. Achim K. Salminen M. Partanen J. (2016). Gata2 and Gata3 regulate the differentiation of serotonergic and glutamatergic neuron subtypes of the dorsal raphe. Development 143, 4495–4508. 10.1242/dev.136614 27789623

[B26] Higashijima S. I. Mandel G. Fetcho J. R. (2004a). Distribution of prospective glutamatergic, glycinergic, and gabaergic neurons in embryonic and larval zebrafish. J. Comp. Neurol. 480, 1–18. 10.1002/cne.20278 15515020

[B27] Higashijima S. I. Schaefer M. Fetcho J. R. (2004b). Neurotransmitter properties of spinal interneurons in embryonic and larval zebrafish. J.Comp. Neurol. 480, 19–37. 10.1002/cne.20279 15515025

[B28] Higashijima S. Masino M. Mandel G. Fetcho J. R. (2004c). Engrailed-1 expression marks a primitive class of inhibitory spinal interneuron. J. Neurosci. 24, 5827–5839. 10.1523/JNEUROSCI.5342-03.2004 15215305 PMC6729218

[B29] Hilinski W. C. Bostrom J. R. England S. J. Juárez-Morales J. L. de Jager S. Armant O.. (2016). Lmx1b is required for the glutamatergic fates of a subset of spinal cord neurons. Neural Dev. 11:16. 10.1186/s13064-016-0070-1 27553035 PMC4995821

[B30] Home P. Kumar R. P. Ganguly A. Saha B. Milano-Foster J. Bhattacharya B.. (2017). Genetic redundancy of GATA factors in the extraembryonic trophoblast lineage ensures the progression of preimplantation and postimplantation mammalian development. Development 144, 876–888. 10.1242/dev.145318 28232602 PMC5374352

[B31] Hubbard J. M. Bohm U. L. Prendergast A. Tseng P. B. Newman M. Stokes C.. (2016). Intraspinal sensory neurons provide powerful inhibition to motor circuits ensuring postural control during locomotion. Curr. Biol. 26, 2841–2853. 10.1016/j.cub.2016.08.026 27720623

[B32] Hutchinson S. A. Eisen J. S. (2006). Islet1 and Islet2 have equivalent abilities to promote motoneuron formation and to specify motoneuron subtype identity. Development 133, 2137–2147. 10.1242/dev.02355 16672347

[B33] Hutchinson S. A. Cheesman S. E. Hale L. A. Boone J. Q. Eisen J. S. (2007). Nkx6 proteins specify one zebrafish primary motoneuron subtype by regulating late islet1 expression. Development 134, 1671–1677. 10.1242/dev.02826 17376808 PMC2586877

[B34] Joshi K. Lee S. Lee B. Lee J. W. Lee S. K. (2009). LMO4 controls the balance between excitatory and inhibitory spinal V2 interneurons. Neuron 61, 839–851. 10.1016/j.neuron.2009.02.011 19323994 PMC2848494

[B35] Juárez-Morales J. L. Schulte C. J. Pezoa S. A. Vallejo G. K. Hilinski W. C. England S. J.. (2016). Evx1 and Evx2 specify excitatory neurotransmitter fates and suppress inhibitory fates through a Pax2-independent mechanism. Neural Dev. 11:5. 10.1186/s13064-016-0059-9 26896392 PMC4759709

[B36] Karunaratne A. Hargrave M. Poh A. Yamada T. (2002). GATA proteins identify a novel ventral interneuron subclass in the developing chick spinal cord. Dev. Biol. 249, 30–43. 10.1006/dbio.2002.0754 12217316

[B37] Kimmel C. B. Ballard W. W. Kimmel S. R. Ullmann B. Schilling T. F. (1995). Stages of embryonic development of the zebrafish. Dev. Dyn. 203, 253–310. 10.1002/aja.1002030302 8589427

[B38] Kimura Y. Okamura Y. Higashijima S. (2006). alx, a zebrafish homolog of Chx10, marks ipsilateral descending excitatory interneurons that participate in the regulation of spinal locomotor circuits. J. Neurosci. 26, 5684–5697. 10.1523/JNEUROSCI.4993-05.2006 16723525 PMC6675258

[B39] Kimura Y. Satou C. Higashijima S. (2008). V2a and V2b neurons are generated by the final divisions of pair-producing progenitors in the zebrafish spinal cord. Development 135, 3001–3005. 10.1242/dev.024802 18684740

[B40] Kobayashi M. Nishikawa K. Yamamoto M. (2001). Hematopoietic regulatory domain of gata1 gene is positively regulated by GATA1 protein in zebrafish embryos. Development 128, 2341–2350. 11493553 10.1242/dev.128.12.2341

[B41] Kok F. O. Shin M. Ni C. W. Gupta A. Grosse A. S. van Impel A.. (2015). Reverse genetic screening reveals poor correlation between morpholino-induced and mutant phenotypes in zebrafish. Dev. Cell 32, 97–108. 10.1016/j.devcel.2014.11.018 25533206 PMC4487878

[B42] Kolmer W. (1921). Das “Sagittalorgan” der Wirbeltiere. Z. Anat. Entwicklungs. 60, 652–717.

[B43] Kurek D. Garinis G. A. van Doorninck J. H. van der Wees J. Grosveld F. G. (2007). Transcriptome and phenotypic analysis reveals Gata3-dependent signalling pathways in murine hair follicles. Development 134, 261–272. 10.1242/dev.02721 17151017

[B44] Lanuza G. Gosgnach S. Pierani A. Jessel T. Goulding M. (2004). Genetic identification of spinal interneurons that coordinate left-right locomotor activity necessary for walking movements. Neuron 42, 375–386. 10.1016/S0896-6273(04)00249-1 15134635

[B45] Lewis K. E. (2006). How do genes regulate simple behaviours? Understanding how different neurons in the vertebrate spinal cord are genetically specified. Philos. Trans. R. Soc. Lond. B Biol. Sci. 361, 45–66. 10.1098/rstb.2005.1778 16553308 PMC1626545

[B46] Lewis K. E. Eisen J. S. (2004). Paraxial mesoderm specifies zebrafish primary motoneuron subtype identity. Development 131, 891–902. 10.1242/dev.00981 14757641

[B47] Lewis K. E. Bates J. Eisen J. S. (2005). Regulation of iro3 expression in the zebrafish spinal cord. Dev. Dyn. 232, 140–148. 10.1002/dvdy.20215 15580554

[B48] Li S. Misra K. Matise M. P. Xiang M. (2005). Foxn4 acts synergistically with Mash1 to specify subtype identity of V2 interneurons in the spinal cord. Proc. Natl. Acad. Sci. U.S.A. 102, 10688–10693. 10.1073/pnas.0504799102 16020526 PMC1180804

[B49] Moran-Rivard L. Kagawa T. Saueressig H. Gross M. K. Burrill J. Goulding M. (2001). Evx1 is a postmitotic determinant of V0 interneuron identity in the spinal cord. Neuron 29, 385–399. 10.1016/S0896-6273(01)00213-6 11239430

[B50] Muller T. Brohmann H. Pierani A. Heppenstall P. A. Lewin G. R. Jessell T. M.. (2002). The homeodomain factor lbx1 distinguishes two major programs of neuronal differentiation in the dorsal spinal cord. Neuron 34, 551–562. 10.1016/S0896-6273(02)00689-X 12062039

[B51] Muroyama Y. Fujiwara Y. Orkin S. H. Rowitch D. H. (2005). Specification of astrocytes by bHLH protein SCL in a restricted region of the neural tube. Nature 438, 360–363. 10.1038/nature04139 16292311

[B52] Nardelli J. Thiesson D. Fujiwara Y. Tsai F. Y. Orkin S. H. (1999). Expression and genetic interaction of transcription factors GATA-2 and GATA-3 during development of the mouse central nervous system. Dev. Biol. 210, 305–321. 10.1006/dbio.1999.9278 10357893

[B53] Okuda Y. Yoda H. Uchikawa M. Furutani-Seiki M. Takeda H. Kondoh H.. (2006). Comparative genomic and expression analysis of group B1 sox genes in zebrafish indicates their diversification during vertebrate evolution. Dev. Dyn. 235, 811–825. 10.1002/dvdy.20678 16408288

[B54] Pai S. Y. Truitt M. L. Ting C. N. Leiden J. M. Glimcher L. H. Ho I. C. (2003). Critical roles for transcription factor GATA-3 in thymocyte development. Immunity 19, 863–875. 10.1016/S1074-7613(03)00328-5 14670303

[B55] Panayi H. Panayiotou E. Orford M. Genethliou N. Mean R. Lapathitis G.. (2010). Sox1 is required for the specification of a novel p2-derived interneuron subtype in the mouse ventral spinal cord. J. Neurosci. 30, 12274–12280. 10.1523/JNEUROSCI.2402-10.2010 20844123 PMC6633433

[B56] Pandolfi P. P. Roth M. E. Karis A. Leonard M. W. Dzierzak E. Grosveld F. G.. (1995). Targeted disruption of the GATA3 gene causes severe abnormalities in the nervous system and in fetal liver haematopoiesis. Nat. Genet. 11, 40–44. 10.1038/ng0995-40 7550312

[B57] Park H. C. Mehta A. Richardson J. S. Appel B. (2002). olig2 is required for zebrafish primary motor neuron and oligodendrocyte development. Dev. Biol. 248, 356–368. 10.1006/dbio.2002.0738 12167410

[B58] Park H. C. Shin J. Appel B. (2004). Spatial and temporal regulation of ventral spinal cord precursor specification by Hedgehog signaling. Development 131, 5959–5969. 10.1242/dev.01456 15539490

[B59] Peng C. Y. Yajima H. Burns C. E. Zon L. I. Sisodia S. S. Pfaff S. L.. (2007). Notch and MAML signaling drives Scl-dependent interneuron diversity in the spinal cord. Neuron 53, 813–827. 10.1016/j.neuron.2007.02.019 17359917 PMC2768132

[B60] Petracca Y. L. Sartoretti M. M. Di Bella D. J. Marin-Burgin A. Carcagno A. L. Schinder A. F.. (2016). The late and dual origin of cerebrospinal fluid-contacting neurons in the mouse spinal cord. Development 143, 880–891. 10.1242/dev.129254 26839365 PMC4813337

[B61] Pillai A. Mansouri A. Behringer R. Westphal H. Goulding M. (2007). Lhx1 and Lhx5 maintain the inhibitory-neurotransmitter status of interneurons in the dorsal spinal cord. Development 134, 357–366. 10.1242/dev.02717 17166926

[B62] Pinheiro P. Gering M. Patient R. (2004). The basic helix-loop-helix transcription factor, Tal2, marks the lateral floor plate of the spinal cord in zebrafish. Gene Expr. Patterns 4, 85–92. 10.1016/S1567-133X(03)00145-5 14678833

[B63] Quan F. B. Dubessy C. Galant S. Kenigfest N. B. Djenoune L. Leprince J.. (2015). Comparative distribution and *in vitro* activities of the urotensin II-related peptides URP1 and URP2 in zebrafish: evidence for their colocalization in spinal cerebrospinal fluid-contacting neurons. PLoS ONE 10:e0119290. 10.1371/journal.pone.0119290 25781313 PMC4364556

[B64] Roberts B. L. Maslam S. Scholten G. Smit W. (1995). Dopaminergic and GABAergic cerebrospinal fluid-contacting neurons along the central canal of the spinal cord of the eel and trout. J. Comp. Neurol. 354, 423–437. 10.1002/cne.903540310 7608330

[B65] Sapir T. Geiman E. J. Wang Z. Velasquez T. Mitsui S. Yoshihara Y.. (2004). Pax6 and engrailed 1 regulate two distinct aspects of renshaw cell development. J. Neurosci. 24, 1255–1264. 10.1523/JNEUROSCI.3187-03.2004 14762144 PMC2997484

[B66] Schäfer M. Kinzel D. Winkler C. (2007). Discontinuous organization and specification of the lateral floor plate in zebrafish. Dev. Biol. 301, 117–129. 10.1016/j.ydbio.2006.09.018 17045256

[B67] Smith E. Hargrave M. Yamada T. Begley C. G. Little M. H. (2002). Coexpression of SCL and GATA3 in the V2 interneurons of the developing mouse spinal cord. Dev. Dyn. 224, 231–237. 10.1002/dvdy.10093 12112475

[B68] Sorrells S. Toruno C. Stewart R. A. Jette C. (2013). Analysis of apoptosis in zebrafish embryos by whole-mount immunofluorescence to detect activated Caspase 3. J. Vis. Exp. 82:e51060 10.3791/51060 PMC4109746 24378359

[B69] Stoeckel M. E. Uhl-Bronner S. Hugel S. Veinante P. Klein M. J. Mutterer J.. (2003). Cerebrospinal fluid-contacting neurons in the rat spinal cord, a gamma-aminobutyric acidergic system expressing the P2X2 subunit of purinergic receptors, PSA-NCAM, and GAP-43 immunoreactivities: light and electron microscopic study. J. Comp. Neurol. 457, 159–174. 10.1002/cne.10565 12541316

[B70] Truett G. E. Heeger P. Mynatt R. L. Truett A. A. Walker J. A. Warman M. L. (2000). Preparation of PCR-quality mouse genomic DNA with hot sodium hydroxide and tris (HotSHOT). Biotechniques 29, 52:54. 10907076 10.2144/00291bm09

[B71] Vigh B. Vigh-Teichmann I. Aros B. (1977). Special dendritic and axonal endings formed by the cerebrospinal fluid contacting neurons of the spinal cord. Cell Tissue Res. 183, 541–552. 10.1007/BF00225666 922853

[B72] Yang L. Rastegar S. Strähle U. (2010). Regulatory interactions specifying Kolmer-Agduhr interneurons. Development 137, 2713–2722. 10.1242/dev.048470 20610488

[B73] Zhang J. Gray J. Wu L. Leone G. Rowan S. Cepko C. L.. (2004). Rb regulates proliferation and rod photoreceptor development in the mouse retina. Nat. Genet. 36, 351–360. 10.1038/ng1318 14991054

[B74] Zhang J. Lanuza G. M. Britz O. Wang Z. Siembab V. C. Zhang Y.. (2014). V1 and v2b interneurons secure the alternating flexor-extensor motor activity mice require for limbed locomotion. Neuron 82, 138–150. 10.1016/j.neuron.2014.02.013 24698273 PMC4096991

[B75] Zhou Y. Yamamoto M. Engel J. D. (2000). GATA2 is required for the generation of V2 interneurons. Development 127, 3829–3838. 10934027 10.1242/dev.127.17.3829

[B76] Zhu C. Smith T. McNulty J. Rayla A. L. Lakshmanan A. Siekmann A. F.. (2011). Evaluation and application of modularly assembled zinc-finger nucleases in zebrafish. Development 138, 4555–4564. 10.1242/dev.066779 21937602 PMC3177320

