# Supplementary material for: Tal1, Gata2a, and Gata3 Have Distinct Functions in the Development of V2b and Cerebrospinal Fluid-Contacting KA Spinal Neurons
Source: Front Neurosci. 2018 Mar 29;12:170. doi: 10.3389/fnins.2018.00170 (PMC5884927; doi:10.3389/fnins.2018.00170)
Supplement: Supplementary file 1 [file DataSheet1.pdf]

Supplementary Material

# Tal1, Gata2a and Gata3 have distinct functions in the development of V2b and cerebrospinal fluid-contacting KA spinal neurons

**L. A. Andrzejczuk*, S. Banerjee*, S. J. England, C. Voufo, K. Kamara and K. E. Lewis§**

*These authors contributed equally to this work. § Corresponding Author: kelewi02@syr.edu

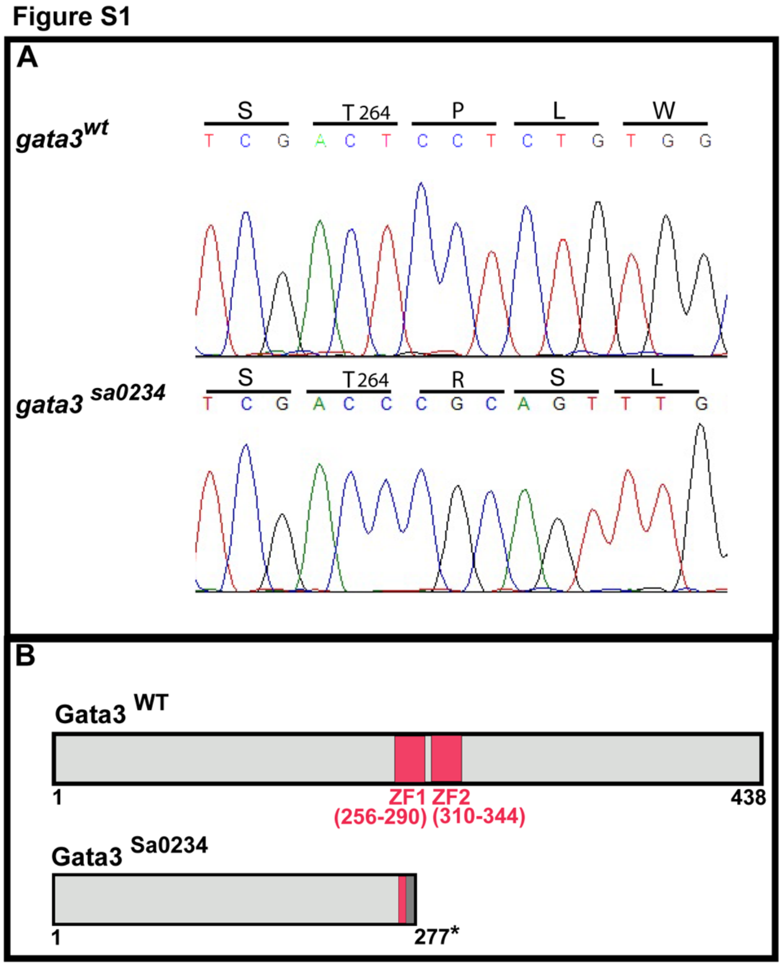


**Supplementary Figure 1**. *gata3*$^{sa0234}$ mutant allele encodes a truncated protein that lacks both zinc finger domains.

(A) Electropherogram of *gata3* WT and *sa0234* mutant alleles with nucleotides, and single letter codes for corresponding amino acids, indicated above each trace. (B) WT Gata3 contains 438 amino acids and two zinc fingers (ZF1 and ZF2) indicated in red. Gata3$^{sa0234}$ protein is truncated after 277 amino acids and has 13 aberrant amino acids (shaded in dark grey) following Threonine 264 (shown in A). Although Gata3$^{sa0234}$ contains the first 8 amino acids of ZF1, EMBL-pfam (pfam.xfam.org/) failed to identify any zinc finger domains in this protein.

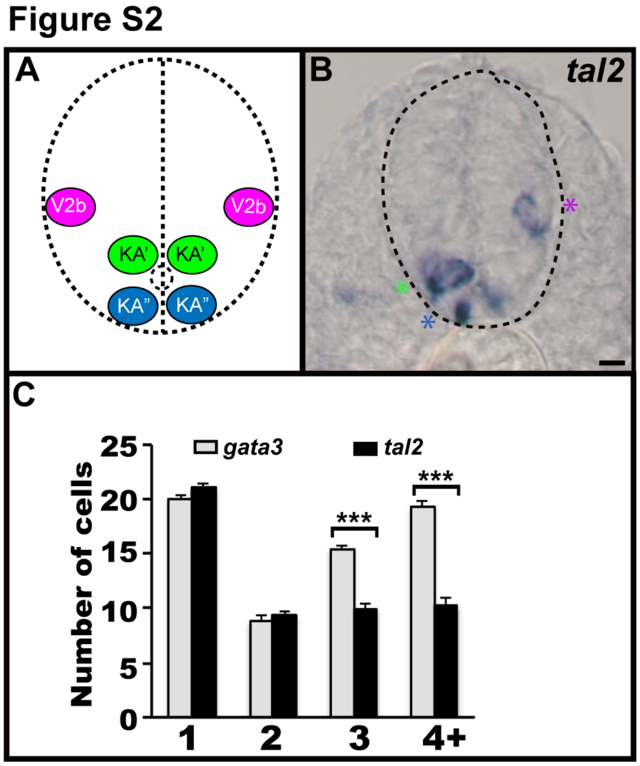


**<u>Supplementary Figure 2</u>**. <u>*tal2* is expressed in all KA neurons and a few V2b neurons.</u>

Cross-sectional (A-B) views of 24h WT zebrafish embryos; dorsal, top. (A) Schematic indicating positions of KA", KA' and V2b neurons. (B) *tal2* expression in KA" (blue asterisk), KA' (green asterisk) and V2b (magenta asterisk) cells. Dotted line (B) shows spinal cord boundary. The spacing / rostral-caudal position of KA'', KA' and V2b cells is not identical on the two sides of the spinal cord. In this particular cross-section there is a V2b cell only on the right hand side and there are more KA neurons on the left hand side than on the right hand side. Scale bar = 10 microns. (C) Mean number of cells expressing *gata3* or *tal2* in each D/V row of spinal cord region adjacent to somites 6-10. All counts are an average of at least 5 embryos. Error bars indicate SEM. Statistically significant ($P<0.001$) comparisons are indicated with square brackets and three stars (***). Compared to *gata3* expression, there are statistically significantly fewer cells expressing *tal2* in row 3 and above, suggesting that *tal2* is only expressed in a subset of V2b cells. For P values see Supplementary Table 3.

Figure S3

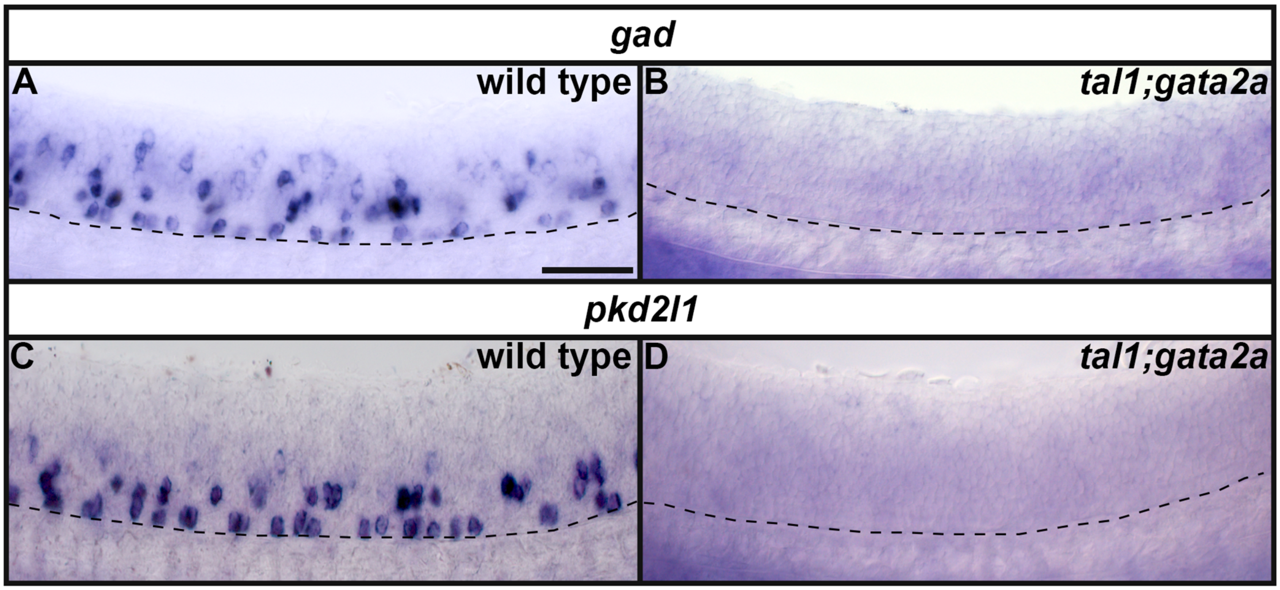


<u>**Supplementary Figure 3**. *gad* and *pkd2l1* expression in *tal1;gata2a* double mutants.</u>

Lateral views of *gad* (A, B) or *pkd2l1* (C, D) expression in 24h WT sibling (A, C) and *tal1;gata2a* double mutant embryos (B, D) as indicated. Dorsal, top; anterior, left. Scale bar = 50 microns. Dashed lines indicate ventral limit of spinal cord.

**Supplementary Table 1.** Primer sequences for PCR *in situ* probe generation.

| Gene | PCR primers |
|---|---|
| *gata2a* | Forward- GTGAGGGTTTCGAGGAGCTC<br>Reverse- **AATTAACCCTCACTAAAGGGA**AGCAACATCGCCTTGGCTAG |
| *gata3* | Forward- CTGCTACCTCCAATCTCCCAC<br>Reverse-***AATTAACCCTCACTAAAGGGA***CAACCATTCAGTCTGCATTACATAAAG |
| *sst1.1* | Forward- AAGATGCTCTCCACGCGTAT<br>Reverse- ***AATTAACCCTCACTAAAGGGA***AGCAGATTTGCACACATTCG |
| *urp1* | Forward- AAGTCAGCCACGATCCTCTCAAC<br>Reverse- ***AATTAACCCTCACTAAAGGGA***AATGAGGGTGTGTTAGGCTGGTC |

PCR primers used to amplify *in situ* hybridization probe templates for genes indicated in the left hand column. In all cases, reverse primers contained the T3 RNA polymerase promoter-binding site (bold italics), used to synthesize antisense RNA probe. Probes were PCR amplified from 27h WT zebrafish cDNA (see materials and methods section in the main paper).

**Supplementary Table 2.** Gene names, previous names, ZFIN identifiers and references for *in situ* hybridization probes used.

| Gene name | ZFIN ID | Reference for probe |
|---|---|---|
| *gata2a* (previously called *gata2*) | ZDB-GENE-980526-260 | This paper (see Supp. Table 1) |
| *gata3* | ZDB-GENE-990415-82 | This paper (see Supp. Table 1) |
| *tal1* (previously called *scl*) | ZDB-GENE-980526-501 | (Peng *et al.*, 2007) |
| *tal2* | ZDB-GENE-040115-1 | (Pinheiro *et al.*, 2004) |
| *sox1a* | ZDB-GENE-040718-186 | (A gift from Strähle lab from library described in Armant *et al*, 2013) |
| *sox1b* | ZDB-GENE-060322-5 | (A gift from Strähle lab from library described in Armant *et al*, 2013) |
| *gad2* (previously called *gad65*) | ZDB-GENE-030909-9 | (Higashijima *et al.*, 2004b&c) |
| *gad1b* (previously called *gad67*) | ZDB-GENE-030909-3 | (Higashijima *et al.*, 2004b&c) |
| *slc17a6a* (previously called *vglut2.2*) | ZDB-GENE-050105-4 | (Higashijima *et al.*, 2004b&c) |
| *slc17a6b* (previously called *vglut2.1)* | ZDB-GENE-030616-554 | (Higashijima *et al.*, 2004b&c) |
| *pkd2l1* | ZDB-GENE-030616-558 | (England *et al.*, 2017) |
| *sst1.1* | ZDB-GENE-030131-4743 | This paper (see Supp. Table 1) |
| *urp1* | ZDB-GENE-100922-138 | This paper (see Supp. Table 1) |
| *sim1a* | ZDB-GENE-020829-1 | (Schäfer *et al.*, 2007) |
| *olig2* | ZDB-GENE-030131-4013 | (Park *et al.*, 2002) |

Column 1 lists genes used in this study, along with previous names where appropriate. Column 2 provides the unique ZFIN identification number for each gene. Column 3 indicates the reference for the *in situ* hybridization probe used in our experiments.

**Supplementary Table 3.** Statistical comparisons of numbers of cells expressing particular genes.

| Figure | Comparison | Gene | Row 1 cells | Row 2 cells | Row 3 cells | Row 4+ cells |
|---|---|---|---|---|---|---|
| 1E | *gata3* vs *tal1* in WT | N/A | 0.539[+] | 0.746[+] | **<0.001**[^] | 0.107[§] |
| 1H | WT vs *tal1* mutant | *gata3* | 0.101[+] | **0.048**[^] | **0.048**[^] | 0.158[+] |
| 1K | WT vs *tal1* mutant | *gata2a* | 0.134[^] | **0.026**[^] | **0.004**[+] | 0.689[+] |
| 1N | WT vs *tal1* mutant | *tal2* | 0.153[+] | **0.020**[^] | **0.020**[^] | **0.020**[^] |
| 3D | WT vs *gata3* mutant | *gata2a* | 0.578[^] | **0.042**[^] | **0.046**[^] | **0.048**[^] |
| 3G | WT vs *gata3* mutant | *tal1* | 0.642[^] | **0.021**[^] | **<0.001**[+] | **0.026**[^] |
| 3J | WT vs *gata3* mutant | *tal2* | 0.420[+] | **0.019**[^] | **0.026**[^] | 0.877[+] |
| 3M | WT vs *gata2a* mutant | *gata3* | **0.017**[^] | 0.550[+] | 0.879[+] | **0.009**[+] |
| 3P | WT vs *gata2a* mutant | *tal1* | **0.027**[^] | 0.298[+] | **0.043**[+] | 0.705[+] |
| 3S | WT vs *gata2a* mutant | *tal2* | 0.056[+] | 0.058[+] | 0.228[+] | 0.611[+] |
| 4D | *gata3* vs *sox1a* in WT | N/A | 0.762[+] | 0.820[+] | **<0.001**[+] | **0.009**[§] |
| 4D | *gata3* vs *sox1b* in WT | N/A | 0.632[+] | 0.795[+] | **0.013**[+] | 0.781[+] |
| 4D | *sox1a* vs *sox1b* in WT | N/A | 0.734[+] | 0.597[+] | 0.787[+] | **0.004**[+] |
| 5D | WT vs *tal1* mutant | *sox1a* | 0.733[+] | **<0.001**[+] | **<0.001**[+] | **0.038**[+] |
| 5G | WT vs *tal1* mutant | *sox1b* | 0.949[+] | **0.044**[^] | **0.001**[+] | **<0.001**[+] |
| 5J | WT vs *gata3* mutant | *sox1a* | 0.792[+] | **0.004**[^] | **0.005**[^] | 0.086[+] |
| 5M | WT vs *gata3* mutant | *sox1b* | 0.920[+] | **0.010**[^] | **0.015**[^] | **0.005**[+] |
| 5P | WT vs *gata2a* mutant | *sox1a* | **0.011**[^] | **0.009**[^] | 0.180[+] | 0.054[^] |
| 5S | WT vs *gata2a* mutant | *sox1b* | 0.320[+] | 0.722[+] | 0.271[+] | 0.754[+] |
| 7C | WT vs *tal1* mutant | *gad* | 0.268[^] | **0.026**[^] | **0.026**[^] | **<0.001**[+] |
| 7F | WT vs *gata3* mutant | *gad* | 1.000[^] | **0.030**[^] | **0.050**[^] | **0.044**[+] |
| 7I | WT vs *gata2a* mutant | *gad* | **<0.001**[+] | **0.026**[^] | 0.586[+] | **0.036**[+] |
| 8E | WT vs *tal1* mutant | *pkd2l1* | 0.125[^] | **0.007**[^] | **0.007**[^] | **0.007**[^] |
| 8F | WT vs *gata3* mutant | *pkd2l1* | 0.181[^] | **0.009**[^] | **0.009**[^] | 0.407[^] |
| 8G | WT vs *gata2a* mutant | *pkd2l1* | **0.010**[^] | 0.327[^] | 0.345[^] | **0.007**[^] |
| 10D | WT vs *gata2a;gata3* double mutants | *gad* | **0.007**[^] | **0.007**[^] | **0.007**[^] | **<0.001**[+] |
| 10D | *gata2a* mutants vs *gata2a;gata3* double mutants | *gad* | **0.042**[^] | **0.009**[^] | **0.010**[^] | **0.002**[+] |
| 10D | *gata3* mutants vs *gata2a;gata3* double mutants | *gad* | **0.010**[^] | 0.371[^] | **0.011**[^] | **<0.001**[+] |
| 10D | *gata2a* mutants vs *gata3* mutants | *gad* | **<0.001**[+] | **0.023**[^] | **<0.001**[+] | **0.001**[+] |
| 10E | WT vs *tal1;gata3* double mutants | *gad* | 0.132[^] | **0.019**[^] | **0.018**[^] | **<0.001**[+] |
| 10E | *tal1* mutants vs *tal1;gata3* double mutants | *gad* | 0.052[^] | 0.608[^] | 0.436[^] | 0.122[+] |
| 10E | *gata3* mutants vs *tal1;gata3* double mutants | *gad* | 0.661[+] | 0.608[^] | **0.037**[^] | **<0.001**[+] |
| 10E | *gata3* mutants vs *tal1* mutants | *gad* | 0.178[^] | 1[^] | **0.017**[+] | **<0.001**[+] |
| 10H | WT vs *tal1;gata3* double mutants | *pkd2l1* | 0.237[^] | **0.007**[^] | **0.007**[^] | **0.010**[^] |

| | | | | | | |
|---|---|---|---|---|---|---|
| 10H | *gata3* mutants vs *tal1;gata3* double mutants | *pkd2l1* | 0.828^ | 0.424^ | 0.424^ | **0.009**^ |
| 10H | *tal1* mutants vs *tal1;gata3* double mutants | *pkd2l1* | 1.000^ | ND | ND | 0.177^ |
| 10H | *tal1* mutants vs *gata3* mutants | *pkd2l1* | 1.000^ | 0.424^ | 0.424^ | **0.006**^ |
| 12C | WT vs *gata2a* mutants | pH3 | 0.374+ | 0.480^ | 0.505^ | 0.653^ |
| 12F | WT vs *gata3* mutants | pH3 | 0.400^ | **0.004**^ | **0.010**+ | **0.012**+ |
| 12I | WT vs *tal1* mutants | pH3 | 0.494+ | **0.026**^ | **0.015**+ | 0.121+ |
| S2C | *gata3* vs *tal2* in WT | N/A | 0.664+ | 0.427+ | <**0.001**^ | <**0.001**+ |

Statistical comparisons between the numbers of cells expressing particular genes. First column indicates the figure that contains the relevant bar chart for the comparison (S = Supplementary Figure). Second column states what the comparison is. Where embryos of different genotypes are being compared, column three states the gene that the comparison refers to. If WT embryos are being compared then N/A (not applicable) is listed in column 3. The last four columns show the P values for the comparisons for each row shown in the corresponding bar chart. Values are rounded up to three decimal places. P values less than 0.001 are listed as <0.001. Statistically significant (P<0.05) values are indicated in bold. Statistical test used is indicated by superscript symbol: Wilcoxon test (Mann-Whitney, ^), type 2 Student's t-test (+) or type 3 Student's t-test (§). For a discussion of why particular tests were chosen see the materials and methods section in the main paper. For double mutant comparisons P values are only listed for comparisons with the double mutants and between the single mutants. Other comparisons can be found in earlier single mutant studies. For figure 10H, P values for rows 2 and 3 could not be determined (ND) as all cell counts were zero in both *tal1* and *tal1;gata3* double mutants.

**Supplementary Table 4.** Cell type specific phenotypes in *tal1* mutants.

| *tal1* mutant | | Row # | 1 | 2 | 3M | 3L | 4+ |
|---|---|---|---|---|---|---|---|
| | | Cells | KA" | KA' | | V2b | |
| *gata3* | WT | Rows | 21.25 | 7.00 | 7.75 | 7.25 | 18.0 |
| | | Cells | 21.25 | 14.75 | | 25.25 | |
| | MUT | Rows | 19.00 | 1.67 | 0.00 | 2.33 | 15.00 |
| | | Cells | 19.00 | 1.67 | | 17.33 | |
| *gata2a* | WT | Rows | 19.50 | 10.25 | 6.75 | 7.25 | 17.5 |
| | | Cells | 19.50 | 17.0 | | 24.75 | |
| | MUT | Rows | 20.25 | 0.25 | 0.00 | 7.00 | 17.0 |
| | | Cells | 20.25 | 0.25 | | 24.00 | |
| *tal2* | WT | Rows | 20.50 | 10.75 | 7.25 | 3.50 | 12.50 |
| | | Cells | 20.50 | 18.00 | | 16.00 | |
| | MUT | Rows | 18.75 | 0.00 | 0.00 | 0.00 | 0.00 |
| | | Cells | 18.75 | 0.00 | | 0.00 | |
| *sox1a* | WT | Rows | 20.25 | 8.25 | 6.75 | 5.25 | 19.25 |
| | | Cells | 20.25 | 15.00 | | 24.50 | |
| | MUT | Rows | 19.75 | 1.25 | 0.00 | 4.50 | 15.25 |
| | | Cells | 19.75 | 1.25 | | 19.75 | |
| *sox1b* | WT | Rows | 20.25 | 11.75 | 9.25 | 3.00 | 13.25 |
| | | Cells | 20.25 | 21.00 | | 16.25 | |
| | MUT | Rows | 20.33 | 0.00 | 0.00 | 2.00 | 1.67 |
| | | Cells | 20.33 | 0.00 | | 3.67 | |
| *gad* | WT | Rows | 21.50 | 12.00 | 7.00 | 6.75 | 30.75 |
| | | Cells | 21.50 | 19.00 | | 37.50 | |
| | MUT | Rows | 21.00 | 0.25 | 0.00 | 0.25 | 12.25 |
| | | Cells | 21.00 | 0.25 | | 12.50 | |
| *pkd2l1* | WT | Rows | 18.00 | 5.80 | 11.40 | 0.60 | 6.80 |
| | | Cells | 18.00 | 17.20 | | 7.40 | |
| | MUT | Rows | 19.20 | 0.00 | 0.00 | 0.00 | 0.00 |
| | | Cells | 19.20 | 0.00 | | 0.00 | |

Gene expression phenotypes in *tal1* mutants. Every other row of the table indicates the mean number of cells expressing specific genes, indicated in the first column, in particular dorsal-ventral spinal cord rows of either WT or *tal1* mutants. Row 3 data is divided into medial (3M) and lateral (3L) cells. Cell type identities associated with cells in particular dorsal/ventral positions, is provided in table header and the number of cells falling into each of these categories is provided in every other row.

**Supplementary Table 5.** Cell type specific phenotypes in *gata3* mutants.

| *gata3* mutant | | Row # | 1 | 2 | 3M | 3L | 4+ |
|---|---|---|---|---|---|---|---|
| | | Cells | KA" | KA' | | V2b | |
| *gata2a* | WT | Rows | 20.75 | 8.00 | 7.00 | 7.00 | 20.25 |
| | | Cells | 20.75 | 15.00 | | 27.25 | |
| | MUT | Rows | 21.33 | 0.00 | 0.00 | 8.67 | 17.67 |
| | | Cells | 21.33 | 0.00 | | 26.34 | |
| *tal1* | WT | Rows | 20.50 | 8.75 | 6.75 | 5.25 | 18.75 |
| | | Cells | 20.50 | 15.50 | | 24.00 | |
| | MUT | Rows | 20.50 | 0.00 | 0.00 | 4.75 | 12.75 |
| | | Cells | 20.50 | 0.00 | | 17.50 | |
| *tal2* | WT | Rows | 21.00 | 10.00 | 6.81 | 3.69 | 11.00 |
| | | Cells | 20.00 | 16.81 | | 14.69 | |
| | MUT | Rows | 20.00 | 0.00 | 0.25 | 0.00 | 10.75 |
| | | Cells | 20.00 | 0.25 | | 10.75 | |
| *sox1a* | WT | Rows | 20.60 | 7.60 | 6.80 | 4.60 | 25.40 |
| | | Cells | 20.60 | 14.40 | | 30.00 | |
| | MUT | Rows | 20.83 | 0.00 | 0.17 | 0.00 | 28.67 |
| | | Cells | 20.83 | 0.17 | | 28.67 | |
| *sox1b* | WT | Rows | 21.25 | 5.50 | 10.77 | 3.48 | 16.75 |
| | | Cells | 21.25 | 16.27 | | 20.23 | |
| | MUT | Rows | 21.40 | 0.00 | 0.20 | 0.00 | 10.40 |
| | | Cells | 21.40 | 0.20 | | 10.40 | |
| *gad* | WT | Rows | 20.00 | 8.33 | 8.67 | 5.33 | 26.33 |
| | | Cells | 20.00 | 17.00 | | 31.66 | |
| | MUT | Rows | 20.25 | 0.00 | 0.00 | 1.75 | 22.50 |
| | | Cells | 20.25 | 0.00 | | 24.25 | |
| *pkd2l1* | WT | Rows | 18.00 | 5.80 | 11.40 | 0.60 | 6.80 |
| | | Cells | 18.00 | 17.20 | | 7.40 | |
| | MUT | Rows | 19.40 | 0.20 | 0.20 | 0.00 | 6.40 |
| | | Cells | 19.40 | 0.40 | | 6.40 | |

Gene expression phenotypes in *gata3* mutants. Every other row of the table indicates the mean number of cells expressing specific genes, indicated in the first column, in particular dorsal-ventral spinal cord rows of either WT or *gata3* mutants. Row 3 data is divided into medial (3M) and lateral (3L) cells. Cell type identities associated with cells in particular dorsal/ventral positions, is provided in table header and the number of cells falling into each of these categories is provided in every other row.

**Supplementary Table 6**. Statistical comparisons of numbers of cells expressing particular genes.

| **Figure** | **Comparison** | **Gene/Protein** | **P value** |
|---|---|---|---|
| 6I | WT (55.2 ±1) vs *tal1* mutants (53.2±1.2) | Islet1/2 | 0.248$^{+}$ |
| 6L | WT (48.8±0.6) vs *gata3* mutants (51±1.4) | Islet1/2 | 0.196$^{+}$ |
| 6O | WT (56.6±1.4) vs *gata2a* mutants (58.3±2) | Islet1/2 | 0.505$^{+}$ |
| 6R | WT (26.3±1.3) vs *tal1* mutants (27.3±1.1) | *vsx1* | 0.582$^{+}$ |
| 6U | WT (28.8±0.5) vs *gata3* mutants (28.5±1.2) | *vsx1* | 0.852$^{+}$ |
| 6X | WT (26.2±0.7) vs *gata2a* mutants (26.8±0.5) | *vsx1* | 0.544$^{+}$ |
| 11C | WT (109.3±2.4) vs *tal1* mutants (112.7±1.2) | *slc17a6a/b* | 0.313$^{+}$ |
| 11F | WT (103.4±1.2) vs *gata3* mutants (107.8±2.9) | *slc17a6a/b* | 0.193$^{+}$ |
| 11I | WT (115±0.6) vs *gata2a* mutants (126.7±2.7) | *slc17a6a/b* | **0.014**$^{+}$ |
| 11L | WT (0±0) vs *gata2a* mutants (10.8±0.6) | *slc17a6a/b* row 1 only | **<0.001**$^{\wedge}$ |
| 11L | WT (0±0) vs *gata2a;tal1* double mutants (11.7±1.2) | *slc17a6a/b* row 1 only | **<0.001**$^{\wedge}$ |
| 11L | WT (0±0) vs *gata2a;gata3* double mutants (9±0.6) | *slc17a6a/b* row 1 only | **<0.001**$^{\wedge}$ |
| 11L | *gata2a* mutants (10.8±0.6) vs *gata2a;tal1* double mutants (11.7±1.2) | *slc17a6a/b* row 1 only | 0.464$^{+}$ |
| 11L | *gata2a* mutants (10.8±0.6) vs *gata2a;gata3* double mutants (9±0.6) | *slc17a6a/b* row 1 only | 0.129$^{+}$ |
| 11P | WT (5±0.4) vs *gata2a* mutants (7.8±0.5) | *sim1a* | **0.017**$^{\wedge}$ |
| 11S | WT (21.4±0.5) vs *gata2a* mutants (30.8±0.9) | *sim1a* | <**0.001**$^{+}$ |
| 13E1 | WT (31.5±1.0) vs *gata3* mutants (36.2±1.4) | pH3 +ve / *olig2* -ve | **0.037**$^{+}$ |
| 13E2 | WT (3±0.4) vs *gata3* mutants (5.6±0.8) | pH3 +ve / *olig2* +ve | **0.026**$^{+}$ |
| 13J1 | WT (30.3±1.3) vs *tal1* mutants (34.5±0.9) | pH3 +ve / *olig2* -ve | 0.069$^{\wedge}$ |
| 13J2 | WT (3.7±0.7) vs *tal1* mutants (7±0.4 ) | pH3 +ve / *olig2* +ve | **0.048**$^{\wedge}$ |
| 13O1 | WT (35.3±2.2) vs *gata3* mutants (39.8±2.8) | pH3 +ve / Nkx6.1 -ve | 0.249$^{+}$ |
| 13O2 | WT (3.5±0.7) vs *gata3* mutants (6.5±0.7) | pH3 +ve / Nkx6.1 +ve | **0.017**$^{+}$ |

Statistical comparisons between mutant and WT embryos. First column indicates the figure panel that contains the relevant bar chart for the comparison. Second column states which genotypes are being compared. Numbers within parentheses indicate mean numbers of cells ± S.E.M. For all cases except 11 L and 6 G-O, cells were counted in all dorsal-ventral spinal cord rows. For 11 L, cells were only counted in row 1. For 6 G-O Islet1/2 positive cells in the two most dorsal rows, which correspond to

Rohon-Beard neurons, were not counted. Only ventral cells that correspond to motoneurons were counted. These also have smaller nuclei than Rohon Beard cells. Column three states the gene or protein or double labeling result that the cell counts and statistical comparison refers to. The last column shows the P value for the comparison. P values are rounded up to three decimal places. Statistically significant ($P<0.05$) values are indicated in bold. Statistical test used is indicated by superscript symbol: Wilcoxon test (Mann-Whitney, $^{\wedge}$), type 2 Student's t-test ($^{+}$) or type 3 Student's t-test ($^{\S}$). For a discussion of why particular tests were chosen see the materials and methods section in the main paper.

**Supplementary Table 7.** Cell type specific phenotypes in double mutants.

| Double mutants | | Row # | 1 | 2 | 3M | 3L | 4+ |
|---|---|---|---|---|---|---|---|
| | | Cells | KA" | KA' | | V2b | |
| *gad* | WT | Rows | 18.80 | 10.20 | 7.20 | 1.80 | 30.60 |
| | | Cells | 18.80 | 17.40 | | 32.40 | |
| | *gata2a* MUT | Rows | 1.75 | 5.75 | 12.50 | 2.25 | 17.50 |
| | | Cells | 1.75 | 18.25 | | 19.75 | |
| | *gata3* MUT | Rows | 17.50 | 0.25 | 1.50 | 2.00 | 27.00 |
| | | Cells | 17.50 | 1.75 | | 29.00 | |
| | *gata2a;gata3* MUT | Rows | 0.00 | 0.00 | 0.00 | 0.00 | 10.60 |
| | | Cells | 0.00 | 0.00 | | 10.60 | |
| *gad* | WT | Rows | 18.80 | 10.20 | 7.20 | 1.80 | 30.60 |
| | | Cells | 18.80 | 17.40 | | 32.40 | |
| | *tal1* MUT | Rows | 19.50 | 0.25 | 0.25 | 0.75 | 6.25 |
| | | Cells | 19.50 | 0.50 | | 7.00 | |
| | *gata3* MUT | Rows | 17.50 | 0.25 | 1.50 | 2.00 | 27.0 |
| | | Cells | 17.50 | 1.75 | | 29.00 | |
| | *tal1;gata3* MUT | Rows | 16.75 | 0.50 | 0.25 | 0.25 | 4.00 |
| | | Cells | 16.75 | 0.75 | | 4.25 | |
| *pkd2l1* | WT | Rows | 18.00 | 5.80 | 11.40 | 0.60 | 6.80 |
| | | Cells | 18.00 | 17.20 | | 7.40 | |
| | *tal1* MUT | Rows | 19.20 | 0.00 | 0.00 | 0.00 | 0.00 |
| | | Cells | 19.20 | 0.00 | | 0.00 | |
| | *gata3* MUT | Rows | 19.40 | 0.20 | 0.20 | 0.00 | 6.40 |
| | | Cells | 19.40 | 0.40 | | 6.40 | |
| | *tal1;gata3* MUT | Rows | 19.00 | 0.00 | 0.00 | 0.00 | 0.40 |
| | | Cells | 19.00 | 0.00 | | 0.40 | |

Gene expression phenotypes in double mutants. Every other row of the table indicates the mean number of cells expressing specific genes, indicated in the first column, in particular dorsal-ventral spinal cord rows of either WTs or mutants. Row 3 data is divided into medial (3M) and lateral (3L) cells. Cell type identities associated with cells in particular dorsal/ventral positions, is provided in table header and the number of cells falling into each of these categories is provided in every other row.
